# Combining genomic prediction and multi-trait indices through stochastic simulations: do index type and deployment order affect genetic gain?

**DOI:** 10.1007/s00122-026-05398-0

**Published:** 2026-09-27

**Authors:** Roberto Fritsche-Neto, Lorena Gabriela Coelho Queiroz, Jesimiel Viana, Kajal Gupta, Kashish Grover, Júlio César DoVale

**Affiliations:** 1 Department of Horticultural Science, North Carolina State University, Kilgore Hall, Raleigh, NC 27607 USA; 2 Department of Biology, Universidade Federal de Lavras, Trevo Rotatório Professor Edmir Sá Santos, s/n, Lavras, 37203-202 MG Brazil; 3 Department of Crop Science, Federal University of Ceará, Fortaleza, Ceará Brazil

## Abstract

Genomic selection (GS) has transformed plant breeding by enabling early selection and potentially reducing cycle length, but how to integrate GS with classical multi-trait selection indices remains unclear. We used stochastic simulations to compare seven strategies combining Smith–Hazel (SH), Pesek–Baker (PB), and empirical (EMP) indices with or without GS in a rice breeding program targeting increased grain yield (GY), reduced chalkiness rate (CR), and stable plant height (PH). Trait importance was set to 70% for GY, 15% for CR, and 15% for PH. After a phenotypic-selection burn-in phase, we simulated ten recurrent cycles and evaluated strategies using population mean, selection accuracy, and additive genetic variance over the years. We also considered scenarios with different breeding cycle lengths (GS-based: 3 years; Traditional: 5 years), but the same selection intensity per stage. Thus, PB provides the best balance in gain responses, in terms of both magnitude and direction, with one of the highest GY, the greatest reduction in CR, and PH stabilization. Accuracy and additive genetic losses were more pronounced when the phenotypically constructed index was used as a single target for genomic prediction, especially for CR and PH. Overall, trait-specific genomic prediction followed by index construction better preserved genetic information and produced responses more consistent with the multi-trait breeding objectives.

**Supplementary Information:**

The online version contains supplementary material available at https://doi.org/10.1007/s00122-026-05398-0.

## Introduction

Plant breeding programs aim to maximize long-term genetic gain while balancing competing objectives such as productivity, stability, and resilience. Because most breeding targets are inherently multi-trait, breeders rarely optimize a single trait in isolation. Instead, breeders must simultaneously improve several traits that may be negatively correlated, while also working within practical limitations such as budget, phenotyping capacity, and shifting market demands. Classical selection index theory, first introduced by Smith (1936) and Hazel (1943), provided the foundation for formalizing multi-trait improvement. By assigning weights proportional to the economic or biological importance of traits, selection indices allow breeders to rank genotypes objectively and reproducibly. This approach helps ensure that improvement in one trait (e.g., yield) does not come at the expense of other critical traits (e.g., disease resistance or end-use quality), thereby supporting balanced genetic progress that aligns with long-term breeding strategies.

The strength of selection indices lies in their flexibility. Weights can be adjusted to reflect industry needs, production environments, or specific market niches (Hazel 1943; Hazel and Lush 1942). Desired-gain indices, for example, enable breeders to set explicit targets for improving or stabilizing traits (Pesek and Baker ), whereas economic indices maximize overall value within a market-based framework. This adaptability makes index construction a critical decision point in a breeding program design. However, despite decades of theoretical development, the realized efficiency of different index types often depends on genetic architecture, trait heritabilities, and trait correlations (Dekkers 2007). Moreover, empirical studies directly comparing alternative index formulations under modern genomic prediction frameworks remain limited.

The integration of genomic selection (GS) has transformed breeding by enabling accurate selection in early developmental stages through genomic estimated breeding values (GEBVs) (Meuwissen et al. 2001; Heffner et al. 2009). By reducing dependence on time-consuming and costly phenotyping, GS accelerates breeding cycles and increases annual genetic gain. However, when comparing GS-based and phenotypic strategies under simulation, it is important to distinguish GS’s operational advantages, such as shorter cycles and reduced phenotyping demands, from the genetic response obtained within a standardized breeding cycle. The predictive power of GS is particularly advantageous for traits with low heritability, traits expressed late in development, or traits that are expensive or destructive to measure (Crossa et al. 2017). When combined with multi-trait selection indices, genomic prediction offers a pathway to achieve simultaneous improvement while reducing phenotyping demands. For example, indices can be calculated directly on GEBVs, allowing breeders to exploit correlations between traits and leverage information from hard-to-measure traits through their genetic relationships with easier-to-measure proxies (Dekkers 2007).

Despite these potential advantages, integrating selection indices and genomic prediction is not trivial. One strategy is to construct an index based on phenotypic data and then apply genomic prediction to the index value as a single synthetic trait (Goddard 2009). Alternatively, GEBVs can first be estimated for individual characteristics and then combined into an index. A third approach integrates the index directly into the genomic prediction model, jointly estimating weights and GEBVs (Akdemir and Isidro-Sánchez 2019). Each strategy may lead to different outcomes depending on trait architecture, the relative accuracy of genomic prediction, and the temporal horizon of breeding goals. For instance, applying genomic prediction before index construction might maximize short-term accuracy, whereas integrating the index within the prediction model may yield more balanced long-term responses. Therefore, understanding how index type and deployment order interact with these underlying genetic parameters is critical for designing strategies that maximize cumulative gain while minimizing the loss of genetic diversity. Furthermore, maintaining genetic variability is particularly important to retain opportunities for future selection in the face of changing environmental and market conditions.

Recently, an empirical study aimed to elucidate the effects of these factors and their interactions (Crossa et al. 2025). For this purpose, the authors evaluated the efficiency of three selection indices combined with genomic prediction using historical rice (Oryza sativa L.) data. Overall, the Smith-Hazel index proved most effective at maximizing key index parameters when applied after genomic prediction. In contrast, the eigenvalue selection index method provides the best predictive accuracy when used alone. However, studies that rely only on historical data have important limitations. They reflect past conditions, which may not represent future environments or market needs, and they may not capture the impact of new breeding tools or technologies. Therefore, conclusions from historical data alone may not always translate well to current or future breeding programs.

In this context, recent advances in stochastic simulation provide an opportunity to evaluate these interactions under controlled scenarios. Simulation allows researchers to systematically vary parameters such as heritability, genetic correlation, and index weights, thereby disentangling the effects of index type and deployment order. Moreover, simulations can assess both short-term responses and long-term consequences, including trade-offs between rapid initial gain and sustained improvement over multiple breeding cycles (Gaynor et al. 2017). By explicitly incorporating stochasticity, such simulations can better mimic the uncertainty inherent in real breeding programs.

In this study, we used stochastic simulations to evaluate how selection index type and alternative architectures for integrating genomic prediction with multi-trait selection indices affect genetic response in a rice breeding program. Specifically, we aimed to (i) quantify how empirical, Smith-Hazel, and Pesek-Baker indices perform when combined or not with genomic prediction; (ii) compare two distinct GS-index integration architectures in which genomic prediction is applied before in trait-specific followed by index construction, and index-traget genomic prediction, in which a phenotypically constructed multi-trait index is used as a single synthetic target for genomic prediction; and (iii) evaluate how these strategies affect population mean, prediction accuracy, and additive genetic variance across recurrent selection cycles. Importantly, we intentionally compared all strategies under the same breeding-cycle length, selection intensity, and population pipeline. Therefore, our objective was to evaluate genetic response per cycle and the consequences of index type and deployment order, rather than annual genetic gain, cost efficiency, or the operational advantages that GS may provide through shorter breeding cycles or reduced phenotyping requirements. By making this distinction, our work provides insights into how GS and selection indices can be integrated to better align multi-trait responses with breeding objectives under controlled simulation conditions.

## Materials and methods

The simulations were developed using the LSU AgCenter rice breeding program as a reference (Silva et al. 2025) and conducted with the AlphaSimR package (Gaynor et al., 2021). In addition, we considered grain yield (GY), chalkiness rate (CR), and plant height (PH) as target traits for improvement. In total, we stochastically simulated seven selection pipelines: traditional strategies based on phenotypic selection (TRAD) using the empirical, Smith-Hazel, and Pesek-Baker multi-trait indices, and genomic selection (GS) strategies using the Smith-Hazel and Pesek-Baker indices. In the traditional pipelines, a five-year breeding cycle was considered, whereas in the GS pipelines, the cycle was reduced to three years.

**Fig. 1:**
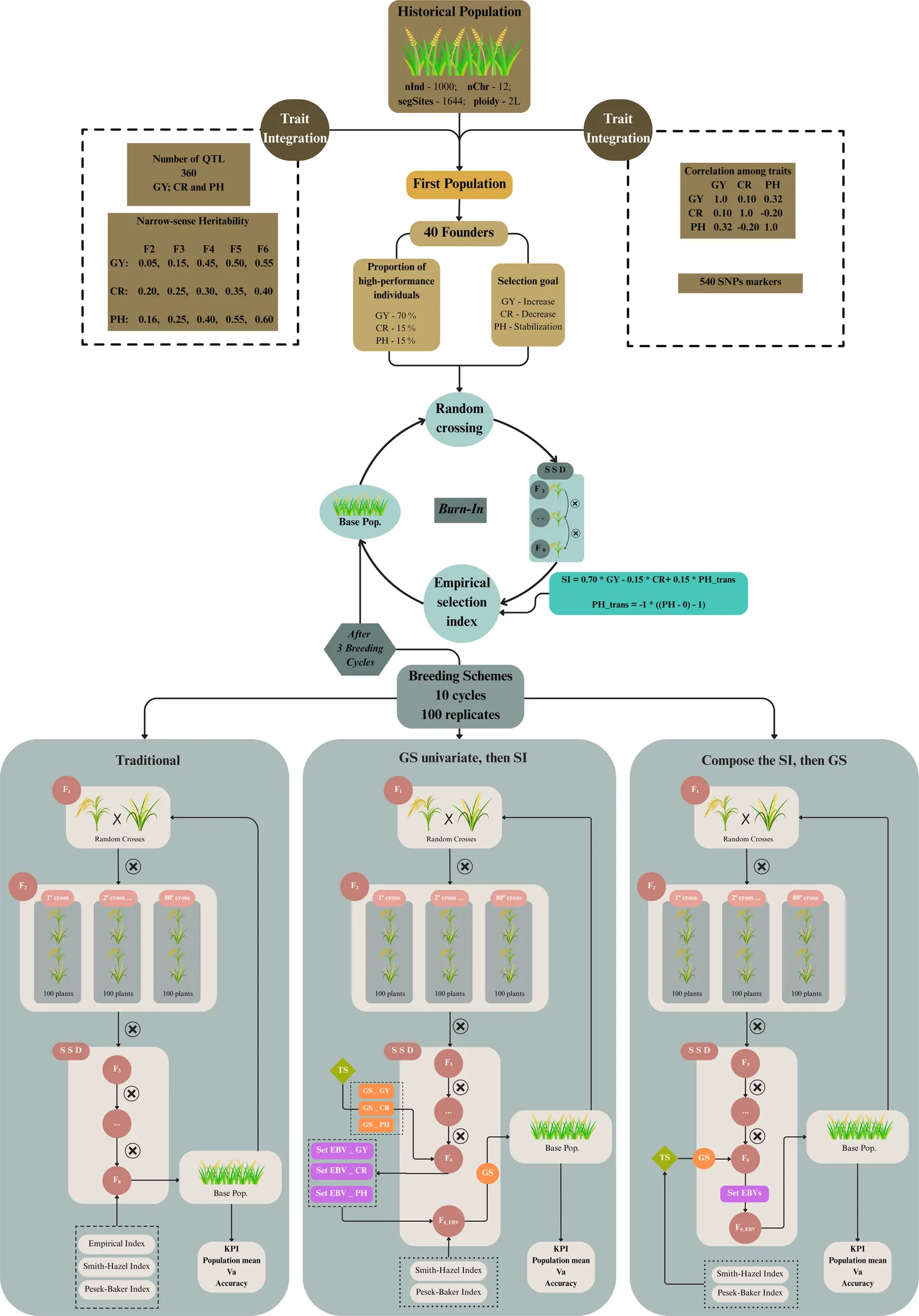
Complete simulation workflow used in this study. The process includes historical population simulation, trait integration, founder selection, burn-in cycles, training set development, breeding program implementation, and final evaluation. Seven distinct selection scenarios were compared at generation F_6_ using empirical (EMP), Smith-Hazel (SH), and Pesek-Baker (PB) selection indices, with or without genomic selection (GS). Scenarios were grouped according to the type and timing of GS application: (i) phenotypic selection only, (ii) GS applied after trait-level prediction (GS–SH, GS–PB), and (iii) GS applied directly on a phenotypic index (SH–GS, PB–GS). Outputs included population mean, accuracy, and additive variance

Although the TRAD (5 years) and GS (3 years) scenarios comprised cycles of different durations, both were designed to evaluate a breeding program over 30 years. This is because the objective of this study was to evaluate how the type of selection index and the timing of genomic selection application would influence accumulated genetic response over time, compared with traditional methods, and not to estimate costs, phenotyping demand, or pipeline operational efficiency. Thus, we could compare the accumulated responses for GY, CR, and PH, since the TRAD and GS scenarios used the same general simulation structure, selection intensity, marker density, genetic architecture, and recurrent selection scheme. In all cases, generation advancement was performed by Single Seed Descent (SSD) up to F_6_, the generation in which selection was applied, with 40 individuals selected per cycle. (Fig. 1).

### Creation of the historical population and base population

The founding population was simulated from an evolutionary history generated with the Markovian coalescent simulator MaCS (Chen et al. 2009), as implemented in the AlphaSimR package (Gaynor et al. 2021). The simulation was initialized using a fixed random seed to ensure reproducibility. A total of 1,000 diploid inbred individuals were generated using 12 chromosomes and 1,644 segregating biallelic sites, corresponding to 137 segregating sites per chromosome. The simulation used the generic species option in AlphaSimR, with no population split and ploidy set to two, representing a simplified self-pollinated rice breeding population.

Three quantitative traits were simulated: grain yield (GY), chalkiness rate (CR), and plant height (PH). For each trait, 360 additive QTLs were simulated, corresponding to 30 QTLs per chromosome. Trait means were set to zero, and additive genetic variances were scaled to one for all traits. No dominance or epistatic effects were included; therefore, all simulated genetic values were determined only by additive effects. The additive genetic correlation structure among traits was imposed directly during trait simulation using the corA argument of the addTraitA() function in AlphaSimR. Table 1 shows the target genetic correlation matrix used for GY, CR, and PH.

**Table 1:** Genetic correlation matrix among grain yield (GY), chalkiness rate (CR), and plant height (PH) used in the simulation scenarios

	GY	CR	PH
**GY**	1.00	0.10	0.32
**CR**	0.10	1.00	-0.20
**PH**	0.32	-0.20	1.00

Additionally, a SNP chip containing 540 markers was simulated using the addSnpChip() function, corresponding to 45 SNP markers per chromosome. These markers were used for genomic prediction in the GS-based scenarios. Environmental variances were defined according to generation-specific heritability values empirically estimated from the rice breeding program used as reference in this study. For the initial population, environmental variance was set using the F_5_ heritability values, and generation-specific heritabilities were subsequently used at different stages of the breeding pipeline, including the burn-in phase and the recurrent selection cycles (Table 2).

**Table 2:** Heritability (h²) values by generation for each simulated trait: grain yield (GY), chalkiness rate (CR), and plant height (PH)

Trait \ Generation	F_2_	F_3_	F_4_	F_5_	F_6_	F_7_	F_8_	F_9_	F_10_
**GY**	0.05	0.15	0.45	0.50	0.55	0.60	0.65	0.70	0.75
**CR**	0.20	0.25	0.30	0.35	0.40	0.45	0.50	0.55	0.60
**PH**	0.16	0.25	0.40	0.55	0.60	0.70	0.85	0.80	0.90

### Selection of parents based on multiple traits

From the initial population (firstPop), the founding parents were selected to start the breeding program. The selection was based on the objectives defined for the selection index, considering the three characteristics described above.

For this purpose, 70% of the individuals were selected based on grain yield, 15% based on the chalkiness rate, and 15% based on plant height, totaling 40 founder individuals. The selection was performed independently for each trait, based on phenotypic values. The individuals selected in each of the three stages were gathered in a list (popList) and combined into a single population using the mergePops function.

To ensure genetic diversity and avoid redundancies, duplicate individuals (based on their IDs) were removed using the unique function. The resulting set, known as founders, served as the basis for subsequent crosses in the program. The additive variance of the founders was evaluated after population consolidation to confirm that genetic variability was maintained among the selected individuals.

### Burn-in phase

The Burn-in simulation was conducted over three breeding cycles, starting with the selected founder population. In each cycle, the 40 parents were randomly crossed, generating 160 crosses, with one plant per cross in F_1_. From F_2_ onwards, the Single Seed Descent (SSD) advancement method was applied, maintaining one progeny per plant until generation F_6_.

During Burn-in, selection was performed in F_6_ based on an empirical multitrait linear index, constructed from the characteristics described. Phenotypic values were standardized internally for each cycle, and plant height was transformed as a function of distance to the target value (zero on the standardized scale). The following formula defined the empirical index (EMP):

$$\begin{aligned} \bf{{EMP}} = \bf{0.70} \times \bf{{GY}}\_\bf{{std}} - \bf{0.15} \times \bf{{CR}}\_\bf{{std}} + \bf{0.15} \times \bf{{PH}}\_\bf{{trans}} \end{aligned}$$

The 40 individuals with the highest index values were selected to form the next-generation population. Next, self-pollination was performed to obtain the F_7_ generation, and a single individual was selected as the final variety based on the phenotypic value. The selected F_6_ population was used as the basis for the next cycle.

At the end of each burn-in cycle, the population genetic mean, the additive variance of each trait, and the average individual performance were recorded to monitor genetic progress.

### Construction of the training set for genomic prediction

After the burn-in cycles, we created a training set to estimate marker effects and calculate genomic breeding values (GEBVs) for the three traits. For this, we performed 80 crosses, each generating 100 progenies, for a total of 8,000 individuals. The heritability used in this step corresponds to the F_4_ generation, definedin the previous table (Table 2). We used the resulting population to estimate the effects of the markers for each trait separately using the RR-BLUP method (Endelman 2011). We also applied the “grandparents-parents-offspring” strategy to reestimate marker effects each breeding cycle, which has been reported as one of the most effective for maintaining GS accuracy over multiple cycles (Sabadin et al. 2022).

Finally, we conducted genomic predictions separately for grain yield (GY), chalkiness rate (CR), and plant height (PH), generating individual GEBVs for each trait. Subsequently, the GEBVs were combined into a single object, representing the multi-trait genomic values of the population.

Prediction accuracy was estimated by the correlation between simulated genetic values and GEBVs for each trait. We also calculated the genetic mean and additive variance for GY, CR, and PH.

### Simulated selection strategies

All evaluated scenarios (Table 3) used the same target traits (GY, CR, and PH). The relative importance assigned to the traits followed the same direction across indices: positive selection for GY, negative selection for CR, and positive selection for PH.

In the traditional methods, for example, the indices were constructed from phenotypic information:

#### Empirical index (TRAD-EMP)

Selection was based on a linear index with fixed weights of 0.70, -0.15, and 0.15 for GY, CR, and PH, respectively. Before calculating the index, the phenotypic values of each trait were standardized within each cycle. For PH, because the objective was not to directly increase or reduce plant height, but to keep it close to the target value, a transformation based on the distance from the target, defined as 0 on the standardized scale, was used. Specifically, PH was transformed as PH_*trans*_ = 1- **|** PH_std_ – PH_*target*_ **|**, where PH_*target*_ = 0. Thus, individuals with PH closer to this value contributed more to the index, whereas larger deviations from the target reduced this contribution.

#### Smith-Hazel index (TRAD-SH)

We used the classical index proposed by Smith (1936) and Hazel (1943). The index was calculated using the phenotypic and genotypic covariance matrices estimated from the F_6_ candidates, along with a vector of breeding objective weights of 0.70, -0.15, and 0.15 for GY, CR, and PH, respectively.

#### Pesek-Baker index (TRAD-PB)

For the Pesek and Baker (1969) index, a vector of desired gains was defined as d = (0.70, -0.15, 0.15)’ for GY, CR, and PH, respectively. These values indicated the direction of selection and the relative importance assigned to each trait. In each cycle, this vector was combined with the phenotypic correlation matrix (R) was estimated from the standardized phenotypic values of the F_6_ candidates, and the index coefficients were obtained as b = R^−1^d. The index value for each candidate was then calculated as I = b’x, where x is the vector of standardized phenotypic values for GY, CR, and PH.

In the scenarios with genomic selection, GS was integrated with the selection indices in two ways: before or after index construction.

#### Trait-specific genomic prediction followed by Smith–Hazel index construction (GS-SH)

Univariate genomic prediction models were fitted via RR-BLUP separately for GY, CR, and PH. The resulting GEBVs were then combined through the Smith–Hazel index, using the same vector of breeding objective weights adopted in the TRAD-SH scenario. Selection was performed based on this index.

#### Smith-Hazel index followed by Genomic Selection (SH-GS)

As in TRAD-SH, the Smith–Hazel index was initially calculated from phenotypic data using weights of 0.70 for GY, -0.15 for CR, and 0.15 for PH. However, after calculating this index, it was used as a single synthetic trait for genomic prediction, and selection was performed based on the predicted genomic values for the index.

#### Trait-specific genomic prediction followed by Pesek-Baker index construction (GS-PB)

The same trait-specific genomic prediction approach was used, but final selection was performed based on the Pesek–Baker index calculated from the GEBVs. For this, the same vector of desired gains adopted in the TRAD-PB scenario was used.

#### Pesek-Baker index followed by Genomic Selection (PB-GS)

Similarly, the Pesek–Baker index was initially calculated from phenotypic data, as described for the TRAD-PB scenario, generating a continuous index value. This index was then used as a single synthetic trait for genomic prediction, and selection was performed based on the predicted genomic values for the index (Table 3).

**Table 3:** Description of the abbreviations used to represent each selection scenario simulated in this study. Scenarios differ in the use of phenotypic or genomic selection and in the type of selection index applied (Smith-Hazel, Pesek-Baker, or empirical)

Abbreviation	Definition
TRAD**-**EMP	Phenotypic selection using the empirical index calculated from standardized phenotypic values
TRAD**-**SH	Phenotypic selection using the Smith-Hazel index calculated from phenotypic trait values
TRAD**-**PB	Phenotypic selection using the Pesek-Baker desired-gain index calculated from phenotypic trait values
GS-SH	Trait-specific genomic prediction followed by Smith-Hazel index construction using GEBVs
GS-PB	Trait-specific genomic prediction followed by Pesek-Baker index construction using GEBVs
SH-GS	Smith-Hazel phenotypic index used as a single synthetic trait for genomic prediction
PB-GS	Pesek-Baker phenotypic index used as a single synthetic trait for genomic prediction

### Key performance indicators

For each scenario, 100 replicates were performed starting from the same post-burn-in population. Thus, each replicate represents an independent stochastic simulation. In all simulated scenarios, the following parameters were estimated: (i) population mean (PM); (ii) additive genetic variance (Va); and (iii) selection accuracy. Calculation methods varied by scenario type.

In traditional scenarios in which selection was based solely on phenotypic information, genetic means and additive genetic variances were estimated from the true genetic values (GVs) of the population selected in F_6_. Selection accuracy was estimated as the square root of the ratio of additive genetic variance to phenotypic variance in the selected population, separately for GY, CR, and PH.

In the GS-SH and GS-PB scenarios, in which genomic prediction was performed separately for each trait before index construction, accuracy was calculated as the correlation between GVs and GEBVs for each trait. The other parameters were extracted from the F_6_ population selected based on the respective index.

In the SH-GS and PB-GS scenarios, in which genomic selection was applied directly to the previously constructed index, accuracy was estimated considering the relationship between the true genetic values of the GY, CR, and PH traits and the predicted genomic values for the synthetic index. Because these scenarios used only the index in the genomic prediction step, this metric served as an auxiliary measure of the index’s ability to discriminate candidates for selection.

The distributions of the results among scenarios were presented using violin plots with superimposed boxplots and trimmed means. Statistical comparisons among methods were performed at the end of the 30-year evaluation window, separately for each trait. For this, the robust Yuen test, based on trimmed means, was used. Pairwise comparisons were adjusted using the Holm method, and differences were considered significant when the adjusted p-value was lower than 0.05.

Finally, all scripts used to perform this study are available in the supplementary material and at https://github.com/rfn-qtl/GS_and_SI.

## Results

### Population mean across selection strategies

**Fig. 2:**
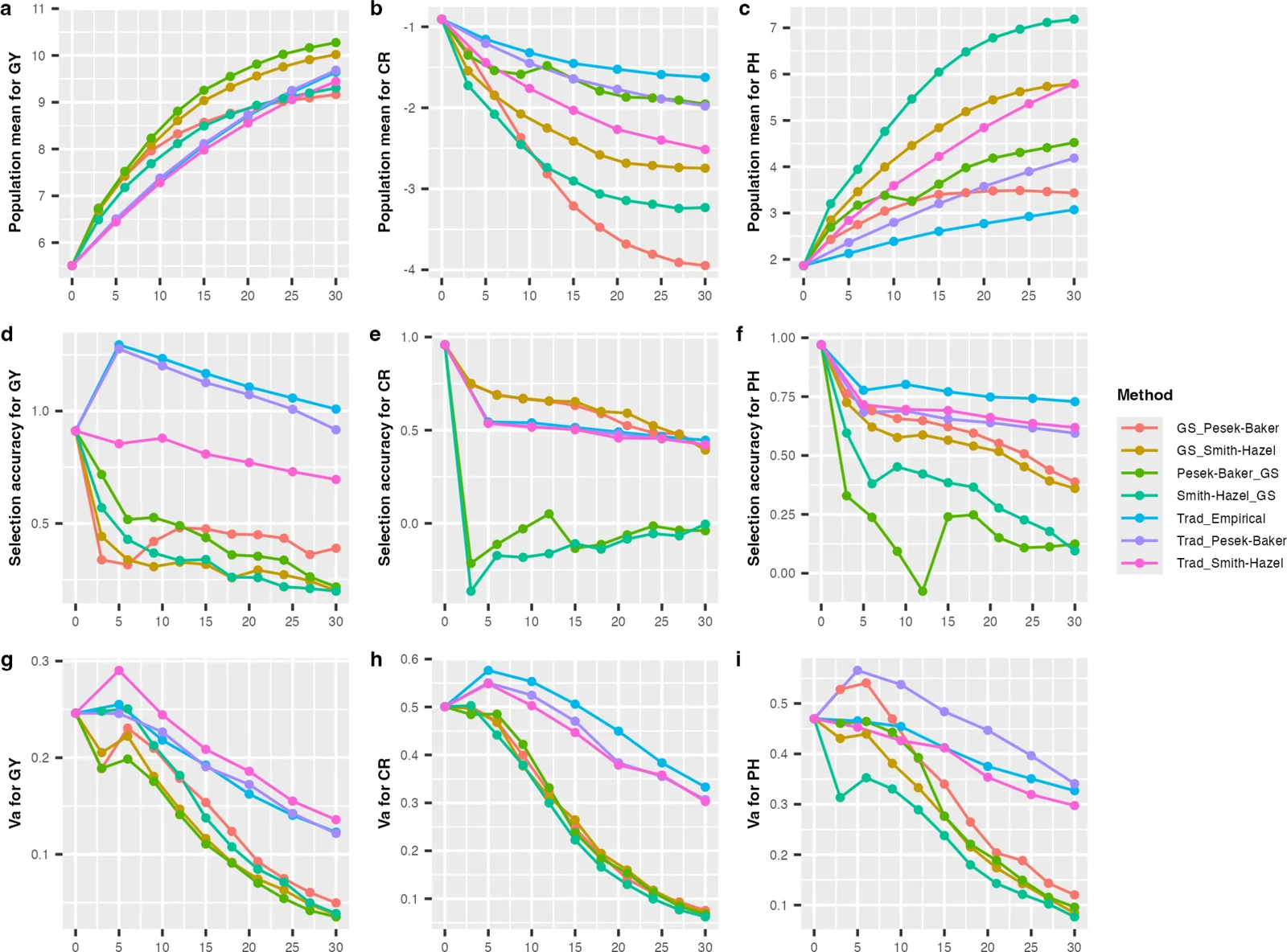
Population means (**a**–**c**), selection accuracy (**d**–**f**), and additive genetic variance (**g**–**i**) for grain yield (GY), chalkiness rate (CR), and plant height (PH) over 30 years of breeding using different combinations of selection indices (Pesek-Baker, Smith-Hazel, and Empirical), and breeding methods (TRAD – Traditional; GS – Genomic Selection)

The evaluation of population means over 30 years of selection revealed marked differences among the evaluated strategies and the analyzed traits (Fig. 2). For GY, the strategies incorporating genomic selection showed the greatest genetic gains at the end of the simulated period. Among them, applying genomic selection directly to the Pesek–Baker index, previously constructed based on phenotypic data, projected the highest gains for GY, with values close to 10.3. In turn, when genomic selection was used to estimate GEBVs for each trait before index construction, the Smith–Hazel index showed the best genetic gain, reaching values close to 10.0 at the end of the evaluation window.

For CR, for which the selection objective was to reduce values across cycles, a distinct pattern was observed. In this case, GS-PB performed best, yielding more negative values close to -4.0, indicating a more pronounced and favorable reduction in CR. In contrast, TRAD-EMP showed values closer to -1.6, suggesting a more moderate selection response for this trait.

Finally, PH also varied among strategies. Scenarios involving the Smith–Hazel index combined with genomic selection, both before and after index construction, yielded the greatest increases in PH. At the end of the 30 simulated years, SH-GS reached values close to 7.1, whereas GS-SH reached approximately 5.7. However, because the objective for PH was to maintain plant height more stably over time, these increases do not necessarily represent the most desirable performance. In this regard, TRAD-EMP showed a more conservative pattern, with less variation across cycles. Similarly, GS-PB also demonstrated medium-term stability, reaching a genetic gain plateau close to 3.5. Although TRAD-EMP showed smaller gains, its trajectory indicated a gradual, linear increase in PH.

### Selection accuracy and additive variance

Selection accuracy generally decreased across cycles, as expected in recurrent selection schemes (Fig. 2). However, this pattern was not uniform across traits and evaluated strategies. For GY, TRAD-EMP and TRAD-PB stood out by showing a marked increase in accuracy over the simulated period. Still within the traditional methods, TRAD-SH showed the most stable pattern, with less fluctuation across cycles. When genomic selection was applied before index construction, GS-PB and GS-SH showed sharper declines in the first cycles. Nevertheless, GS-PB showed a slight subsequent recovery, followed by a phase of relative stability. In contrast, when genomic selection was applied directly to the previously constructed indices, PB-GS and SH-GS maintained a continuous pattern of accuracy reduction as selection advanced.

In CR, accuracy decreased across all strategies, though to different extents. In the traditional methods and in the approaches where genomic selection preceded index construction, this loss occurred mainly in the initial cycles and was followed by a period of stabilization. Within this group, the smallest reductions were observed when genomic selection was applied before index construction, whereas traditional methods showed an intermediate pattern. In contrast, PB-GS and SH-GS exhibited a more abrupt decline, reaching values close to zero already in the first breeding cycle. After this initial drop, accuracy recovered slightly, but remained low in subsequent cycles. Between these two scenarios, SH-GS showed a more regular pattern after recovery, whereas PB-GS followed a more unstable trajectory, alternating small increases with further reductions.

Regarding PH, the pattern was similar to that observed for CR, with the greatest losses in accuracy occurring when genomic selection was applied directly to the indices, especially in PB-GS. Conversely, the traditional methods showed an initial reduction followed by stabilization, suggesting lower sensitivity of accuracy to advances in selection cycles. Strategies in which genomic selection was applied before index construction also followed a pattern similar to that of the traditional methods; however, this stability was maintained only in the medium term, after which slight reductions were again observed.

In addition to accuracy, additive genetic variance (Va) also showed distinct responses across traits. In GY, the traditional method based on the Smith–Hazel index showed an increase in Va in the first cycle, whereas the other traditional methods remained relatively stable during the same period. From the following cycles onward, however, Va gradually decreased in these scenarios. In the strategies involving genomic selection, the reduction was more pronounced, regardless of whether GS was applied before or after index construction. Although the overall patterns of decline were similar, GS-SH and PB-GS showed the greatest depletion of additive genetic variance.

For CR, the dynamics of Va were similar to those observed for GY. The traditional methods showed an initial increase, followed by reductions in subsequent cycles. By contrast, the genomic selection strategies maintained Va relatively stable at the beginning but later showed a more pronounced decline as selection advanced. For PH, SH-GS showed the greatest reduction, whereas traditional methods showed less abrupt losses. GS-PB also increased Va in the first cycle, followed by a progressive reduction over the remaining cycles.

### Genomic vs. Traditional selection

**Fig. 3:**
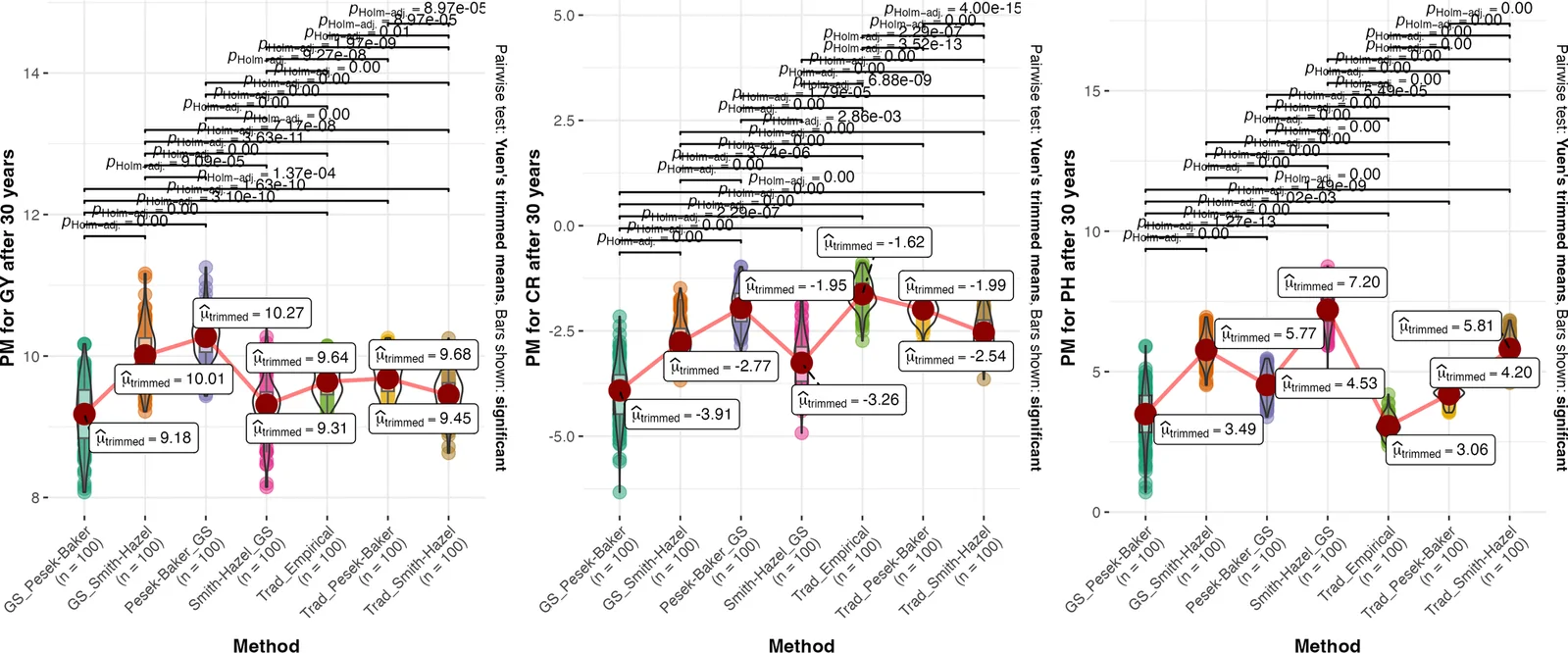
Population means (PM) for grain yield (GY), chalkiness rate (CR), and plant height (PH) after 30 breeding years across different selection methods under genomic selection (GS) and traditional (TRAD) breeding. Statistical comparisons among methods were performed using Yuen’s robust test based on trimmed means. Pairwise comparisons were adjusted using the Holm method, and differences were considered significant when the adjusted p-value was lower than 0.05

The predicted population means at the end of the simulated period showed that the outcomes were strongly influenced by both the selection index and the breeding method adopted (Fig. 3). For GY, PB-GS achieved the highest predicted values, close to 10.3, followed by GS-SH, with a mean close to 10.0. TRAD-EMP and TRAD-PB showed similar performance, with values around 9.6, whereas GS-PB had lower predicted means, around 9.2. In CR, GS-PB produced the most negative means, close to -3.9, indicating a greater reduction in this trait. For PH, SH-GS led to the highest values, reflecting a greater increase in plant height. Conversely, GS-PB and TRAD-EMP showed the smallest increments in PH, with gains of 3.5 and 3.1, respectively. Overall, the statistical tests confirmed significant pairwise contrasts among most of the evaluated methods.

**Fig. 4:**
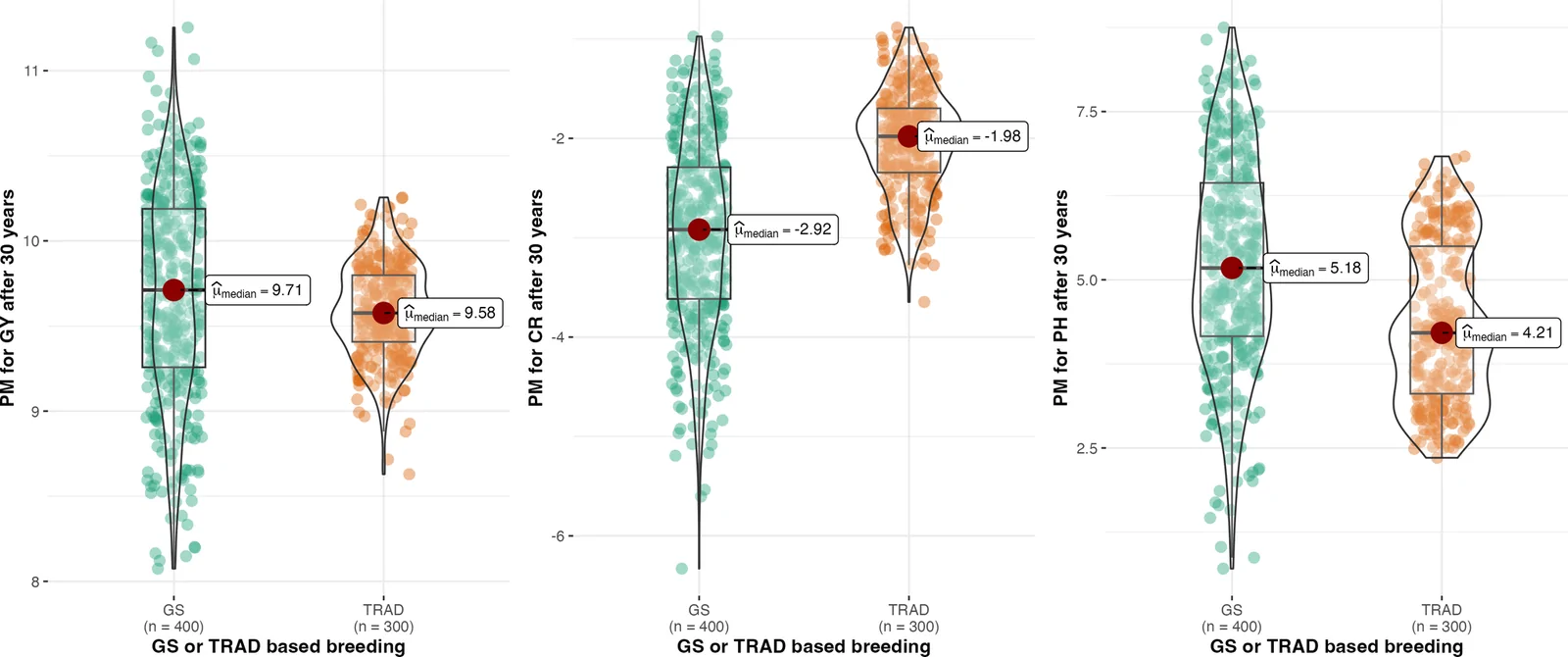
Population means (PM) for grain yield (GY), chalkiness rate (CR), and plant height (PH) after 30 breeding years using genomic selection and traditional breeding

After grouping the strategies into TRAD and GS approaches, numerical differences remained, although they were not statistically significant between groups (Fig. 4). For GY, both groups showed very similar means, around 9.6 for TRAD and 9.7 for GS. In CR, TRAD values were close to -2.0, whereas GS values were approximately − 2.9, indicating a greater reduction in CR with the incorporation of genomic selection. For PH, the GS methods showed higher mean values, close to 5.2, while the TRAD methods remained around 4.2.

### Influence of selection index type

**Fig. 5:**
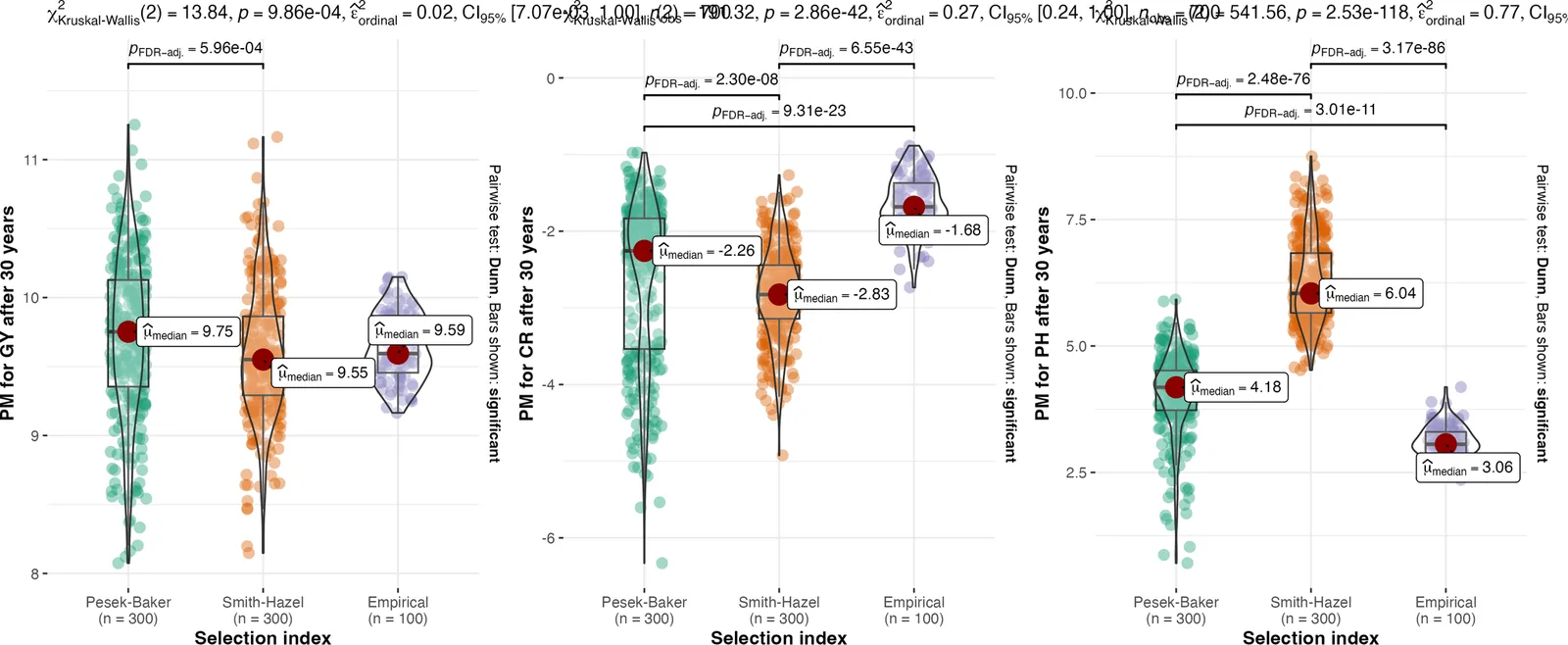
Population means (PM) after 30 breeding years for grain yield (GY), chalkiness rate (CR), and plant height (PH) under three selection indices: Pesek-Baker, Smith-Hazel, and Empirical. Statistical comparisons among methods were performed using Yuen’s robust test based on trimmed means. Pairwise comparisons were adjusted using the Holm method, and differences were considered significant when the adjusted p-value was lower than 0.05

Pairwise comparisons confirmed significant differences for most contrasts among selection indices based on the population means of the three evaluated traits (Fig. 5). For GY, the Pesek–Baker index had the highest median, around 9.8, followed by Smith–Hazel, with a median around 9.6. For CR, where the desired direction was to reduce values across cycles, the Smith–Hazel index performed best, with the lowest population median, close to -2.8. The Pesek–Baker index also promoted a reduction in CR, with a median around − 2.3, whereas the empirical index showed a more moderate response, with values close to -1.7. Finally, for PH, the Smith–Hazel index produced the highest values, with a median close to 6.0. However, given the objective of maintaining PH more stably, the Pesek–Baker index showed an intermediate pattern, with a mean close to 4.2, while the empirical index produced the lowest values, around 3.1.

### Order of GS application relative to index construction

**Fig. 6:**
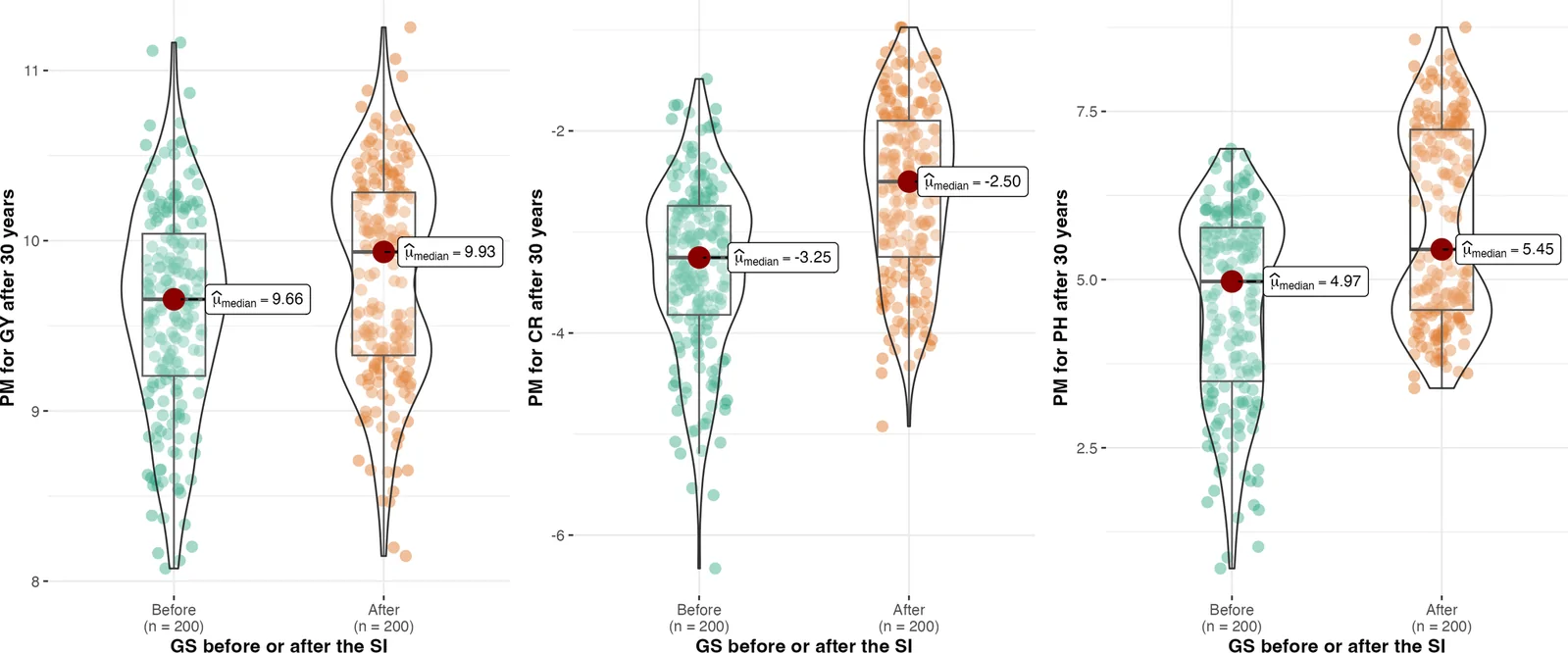
Predicted means (PM) for grain yield (GY), chalkiness rate (CR), and plant height (PH) after 30 breeding years when genomic selection (GS) was applied before or after the selection index (SI)

Considering only strategies that incorporated genomic selection, the position of GS relative to index construction influenced the population means of GY, CR, and PH differently (Fig. 6). Although the differences were not significant according to the paired test, the projected means for GY were numerically higher when genomic selection was applied directly to the previously constructed index, reaching a value close to 9.93. For CR, the direction of the response was different. Applying genomic selection before index construction resulted in a greater reduction in this trait, with a mean close to -3.3, compared with applying GS after index construction, which showed a mean close to -2.5. A similar trend was observed for PH. Considering the objective of maintaining plant height more stable over time, applying GS before index construction showed a more favorable response, with a mean close to 5.0, lower than that observed when genomic selection was applied after index construction, which reached values close to 5.5.

## Discussion

This study provides evidence on how genomic selection (GS) interacts with classical selection indices (SI) to shape multi-trait improvement in simulated rice breeding scenarios. Our results demonstrate that neither GS nor traditional phenotypic selection (TRAD) is universally superior; instead, their relative performance depends on the trait architecture and the breeder’s objectives. These findings align with the view that multi-trait breeding is inherently complex and requires a balance between short-term responses and long-term sustainability (Hazel 1943; Bernardo 2020; Lin et al. 2022).

### Genomic vs. traditional selection

Increasing grain yield remains one of the main goals of rice breeding, especially in the face of climate change and population growth, which increase pressure on the crop production chain (Peng et al. 2008; Seck et al. 2012; Khush 2013). However, under a multi-trait selection objective, higher gains for GY are not sufficient to define the best pipeline. This occurs because the ideotype considered in this study also involves reducing chalkiness rate (CR) and maintaining plant height (PH) at agronomically adequate levels. For CR, for example, the desired direction of selection is opposite that of GY, since higher chalkiness compromises the milling, consumption, and cooking properties of the grains (Sultana et al. 2022). For PH, in turn, the interest is not in promoting marked changes in the current height present in the population, but in avoiding undesirable increases, since excessively tall plants tend to be more susceptible to lodging, with negative effects on the final stand, management, and crop productivity (Wu et al. 2024; Liu et al. 2024).

For this reason, using selection indices in our simulations matters because they strategically direct genetic response toward the objectives of the breeding program, rather than simply maximizing a single trait. In our results, we observed that, in scenarios incorporating genomic selection, projected gains in GY were more pronounced than in traditional scenarios over 30 years, reinforcing GS’s potential to increase selection efficiency.

However, the gains from applying GS were not uniform across pipelines. This is because the response varied by index and by when genomic predictions were used in the selection process. GS, when applied directly to the Pesek–Baker index, resulted in the greatest gains for GY, whereas for the Smith–Hazel index, applying GS before index construction produced a more favorable response. Therefore, GS should not be interpreted only as a superior alternative to traditional selection, but as a tool whose efficiency depends on the way it is integrated into the selection criterion.

Additionally, although GS showed an advantage for GY, interpreting the scenarios must account for correlated responses in CR and PH, since in some pipelines genomic selection also contributed to reductions in CR, with more pronounced responses in PH. Thus, the main result is not only that GS increased genetic gain, but also that its effectiveness in a multi-trait program depends on its ability to combine higher productivity with reduced chalkiness and stable plant height.

### Trade-offs among selection indices

Observing the objectives stipulated by the breeding program that we simulated, that is, increasing GY, reducing CR, and maintaining PH close to the current level, we were able to demonstrate, in general, the complexity associated with multi-trait selection, especially in scenarios in which the traits present distinct correlations among themselves. Part of this is due to the differential results observed with the different selection indices evaluated, since these, depending on the trait, generated distinct responses in direction and magnitude.

Among the indices evaluated, the empirical index stood out as one of the greatest drivers of average gains for GY. This result was expected, since this trait had the highest relative weight among those evaluated, and the index provided greater genetic gains for it. However, although we observed the index’s effectiveness for GY, the reductions were smaller for CR. For PH, although the response was positive, this index contributed to less pronounced increases, which may be favorable given the need to maintain little variation in plant height throughout the selection cycles.

For the Smith–Hazel index, we observed intermediate gains in GY and more evident reductions in CR, indicating that this index responded well while balancing productivity and grain quality. The negative point, however, was the response in PH. This index projected a more pronounced increase in plant height, which does not align with the study’s objective, since the intention was to keep PH relatively stable across cycles. This behavior shows that, even though the index combines different traits based on variance-covariance matrices and economic weights (Smith 1936; Hazel 1943), the final response may shift when trait relationships favor indirect gains in a trait that is not intended to increase.

On average, Pesek–Baker was the most consistent with the previously defined objectives, especially when combined with genomic selection. This strategy resulted in one of the greatest reductions in CR, smaller undesirable changes in PH, and expressive gains for GY. This response is coherent with the nature of the Pesek–Baker index, which is based on desired gains (Pesek and Baker 1969), and may be more adequate when the breeding objective is not simply to maximize productivity, but to direct selection toward a specific ideotype.

Based on our results, choosing the best pipeline only by the greatest gain in GY may lead to an incomplete interpretation. When the program’s objective also involves reducing CR and maintaining PH close to the current level, the index’s performance should be evaluated across the set of responses, not an isolated trait. In this sense, selection indices are useful precisely because they allow different program goals to be combined into a single selection criterion (Pallavi et al. 2024). Among the strategies evaluated, combining genomic selection with the Pesek–Baker index best aligned with this objective, as it maintained relevant gains in GY, favored reduced CR, and avoided more pronounced changes in PH. This result is important because reducing CR remains a challenging goal in rice breeding, both because of the trait’s genetic complexity and its potential association with other agronomic traits (Chen et al. 2024).

### Order of GS relative to index construction

In our results, applying genomic selection to estimate GEBVs for each trait proved, on average, more aligned with the objectives previously established by the breeding program, namely, to reduce CR and maintain PH at more stable levels. However, although this strategy favored more coherent responses for these two traits, it also resulted in a slight penalty in expected gains for GY, especially compared with strategies in which genomic selection was applied directly to the previously constructed index. Even so, given that CR poses a significant challenge for breeding programs, applying GS before index construction appears to be a more consistent alternative with the program’s objectives, even though the gains in GY are numerically lower.

Part of this trend may relate to the structure of the indices under evaluation. One possible explanation is that, in these scenarios, the indices were constructed from estimated genomic values for each simulated trait. In other words, univariate genomic prediction models were initially fitted via RR-BLUP for GY, CR, and PH, and the resulting GEBVs were subsequently combined into a selection index. This strategy is consistent with the logic of linear genomic indices, in which the index is formed by combining GEBVs to predict the aggregate genetic merit of each individual, rather than being constructed directly from observed phenotypes (Ceron-Rojas et al. 2015; Cerón-Rojas and Crossa 2019). In this way, the index tends to operate on values that are closer to the genetic component of each trait, reducing the influence of environmental noise on selection decisions.

In addition, the results indicate that not all indices responded similarly to this strategy. Among the evaluated indices, Pesek–Baker was more aligned with the program’s expected response direction, especially because it favored simultaneously reducing CR and maintaining PH without markedly compromising gains in GY. This result suggests that the efficiency of integrating genomic selection with multi-trait indices depends not only on the stage at which GS is applied, but also on the mathematical structure and assumptions underlying each index.

On the other hand, when genomic selection is applied directly to an index previously constructed on phenotypic data, some specific information for GY, CR, and PH may be compressed into a single value before the prediction step. This simplification may limit the genomic model’s ability to capture the specific genetic architecture of each trait, especially when traits differ in heritability, genetic correlations, and the direction of selection response. In addition, an index initially calculated from phenotypes may incorporate environmental effects and phenotypic correlations that do not always accurately reflect the true genetic relationships among traits.

Thus, our results suggest that constructing selection indices from individuals’ estimated genomic values may lead to more consistent decisions with the objectives of the breeding program. This approach allows the specific genetic information of each trait to be exploited before combining them into an aggregate selection criterion, potentially increasing the efficiency with which simultaneous gains in productivity, grain quality, and plant architecture are achieved.

### Selection accuracy and additive variance

Selection accuracy generally decreased across simulated cycles, as expected in recurrent selection schemes. As superior individuals are repeatedly selected, the frequency of favorable alleles increases, and a portion of the available additive genetic variance is converted into genetic gain across cycles (Viana et al. 2026). Consequently, the population tends to become more genetically uniform, reducing contrast among selection candidates and making genotype ranking progressively more challenging. However, this study shows that this general explanation is insufficient to account for all observed patterns, as accuracy dynamics varied across traits and across strategies used to integrate genomic selection and selection indices.

For GY, for example, the traditional scenarios TRAD-EMP and TRAD-PB showed an expressive increase in accuracy across cycles, whereas TRAD-SH maintained a more stable pattern. This result indicates that, for grain yield, selection based on traditional phenotypic indices still maintained, or even increased, the ability to discriminate superior genotypes over time. This may be because GY had the highest relative weight in the selection objective, leading the traditional indices to better align with the expected direction of gain for this trait. In contrast, when genomic selection was applied before index construction, GS-PB and GS-SH showed more pronounced reductions in the initial cycles, though GS-PB subsequently recovered and then stabilized. In scenarios where genomic selection was applied directly to the previously constructed indices, PB-GS and SH-GS maintained a continuous trajectory of accuracy reduction, suggesting these models were less able to preserve predictive power as selection progressed.

For CR and PH, the pattern was even more evident. In CR, accuracy decreased in all strategies, but the greatest losses occurred when genomic selection was applied directly to the indices, especially in PB-GS and SH-GS, which reached values close to zero already in the first breeding cycle. This result suggests that, when applying genomic prediction to a previously aggregated index, some of the specific information associated with CR reduction may have been lost or diluted before the prediction step. Since CR represents a trait whose selection direction is opposite to that of GY, that is, its reduction is desired while productivity is expected to increase, the compression of information into a single index may have made it difficult to capture the specific genetic architecture of this trait. Although a slight subsequent recovery occurred, accuracy remained low in subsequent cycles, indicating that the initial loss of discriminatory power persisted and reduced selection efficiency for this trait.

A similar behavior was observed for PH. The greatest accuracy losses occurred when genomic selection was applied after index construction, particularly in PB-GS. Because the objective for PH was not to strongly increase or decrease the trait, but rather to maintain it at desirable levels, this result underscores the difficulty of working with multi-trait objectives when traits exhibit distinct selection directions. In this case, applying genomic selection directly to an aggregated index may have limited the model’s ability to capture specific deviations associated with PH stability. In contrast, traditional methods and strategies that applied genomic selection before index construction showed an initial decrease followed by greater stability, although small reductions occurred again in the medium term.

The dynamics of additive genetic variance also help to interpret these results, but should not be seen as the only explanation for changes in accuracy. Likewise, greater residual additive genetic variance indicates greater retention of genetic variability but should not be interpreted alone as evidence of greater future genetic gain. For GY, Va decreased more markedly in the genomic selection scenarios, regardless of whether GS was applied before or after index construction, with greater depletion in GS-SH and PB-GS. For CR, the traditional methods showed an initial increase in Va, followed by reductions in subsequent cycles. In contrast, the strategies with genomic selection maintained Va relatively stable at the beginning but showed a more pronounced decline as selection progressed. For PH, SH-GS showed the greatest reduction in Va, whereas the traditional methods showed less abrupt losses. These results indicate that the relationship between accuracy and Va was not uniform across traits and strategies. In some cases, the reduction in accuracy appeared to be associated with the loss of genetic variability; in others, especially for CR and PH in the PB-GS and SH-GS scenarios, the abrupt decline in accuracy was so intense that it also suggests a methodological limitation related to the way in which the multi-trait information was presented to the genomic model.

In this sense, the main difference among the strategies lies in the stage at which the genetic information of each trait was explored. When genomic selection was applied after index construction, the model predicted an aggregate value previously calculated from the combination of different traits. Although this approach may be efficient when the index accurately reflects the total genetic merit of individuals, it may also reduce trait-specific information before genomic prediction. This is particularly problematic in multi-trait contexts, in which GY, CR, and PH have different weights, selection directions, and possibly distinct heritabilities and genetic correlations. Thus, by condensing this information into a single value before prediction, the model may have lost some of its ability to capture the specific genetic signals underlying the reduction of CR and the maintenance of PH.

By contrast, when genomic prediction was performed before index construction, the models were separately estimated for GY, CR, and PH, allowing GEBVs to be estimated for each trait before combining them into an aggregate selection criterion. This approach has better preserved the trait-specific genetic information, especially for CR and PH, resulting in less severe reductions in accuracy and greater stability after the initial cycles. This result is consistent with the logic of linear genomic indices, in which the genomic values estimated for each trait are combined to predict the aggregate genetic merit of individuals rather than directly predicting an already condensed index (Ceron-Rojas et al. 2015; Cerón-Rojas and Crossa 2019).

Furthermore, updating the training population across generations may have helped avoid even more pronounced accuracy losses in strategies that performed genomic prediction before index construction. By including individuals more genetically related to the selection candidates, this updating tends to maintain greater connectivity between the training and prediction populations, a fundamental factor for the efficiency of recurrent genomic selection (Sabadin et al. 2022). Even so, the results show that simply updating the training population is not sufficient to guarantee stable accuracy when trait-specific information is previously compressed into an aggregate index.

Therefore, our results indicate that the efficiency of integrating genomic selection with selection indices depends strongly on the integration architecture evaluated. Applying genomic selection directly to previously constructed indices resulted in greater reductions in accuracy, especially for CR and PH. It was also associated with a greater reduction in additive genetic variance in some scenarios. In contrast, estimating the genomic values for each trait before index construction enabled the specific genetic information for GY, CR, and PH to be explored before combining them into an aggregate criterion. This strategy better aligned with the breeding program’s objective of simultaneously increasing GY, reducing CR, and maintaining PH at desirable levels. Thus, beyond the magnitude of the cumulative genetic response, the results reinforce that the stage at which genomic prediction is applied can directly influence the direction and consistency of the response to selection in multi-trait breeding programs.

### Implications for breeding programs

Selection indices provide breeders with a powerful tool to simultaneously improve multiple traits of economic and agronomic importance. By combining phenotypic and genetic information into a weighted index, breeders can make decisions that reflect not only the biological correlations among traits but also the specific objectives of a breeding program (Hazel 1943; Bernardo 2020). Importantly, assigning different weights allows breeders to target traits for increase, decrease, or even stabilization, depending on production goals and market requirements. For example, yield or disease resistance are typically selected for increase, whereas traits such as plant height or susceptibility are often targeted for reduction (Falconer and Mackay 1996).

In certain crops, however, some quality traits must be maintained within a narrow range rather than shifted toward an extreme. Malting barley is a key example: while breeders seek to increase yield and disease resistance, grain quality parameters such as protein content, diastatic power, and kernel size must fall within strict thresholds to meet brewing industry standards (Fox et al. 2003; Molina-Cano et al. 1997). Protein content that is too high reduces extract yield and impairs beer stability, while protein content that is too low compromises enzyme activity essential for malting (Eagles et al. 1995). As such, stabilizing selection is applied to keep these traits within an acceptable range rather than driving directional improvement. This illustrates how selection indices can accommodate diverse breeding goals, balancing gains across traits while avoiding detrimental shifts in quality attributes.

In our simulations, the advantage of integrating genomic selection with selection indices became clearer when we evaluated genetic responses against the breeding objective defined for the three traits. Overall, GS-based strategies produced more favorable responses than TRAD scenarios, particularly when genomic prediction was performed before index construction. This order of implementation allowed trait-specific genomic values for GY, CR, and PH to be estimated first and then combined according to the desired selection weights. Among the evaluated strategies, the GS-Pesek-Baker combination showed the most favorable balance, increasing genetic gain for GY while strongly reducing CR and avoiding undesirable shifts in PH. These results indicate that, in multi-trait breeding objectives with different selection directions, the stage at which GS is integrated into the selection index can influence not only the magnitude of genetic gain but also the direction of the genetic response.

Taken together, these findings highlight that GS should not be viewed only as a replacement for traditional selection, but rather as a tool whose efficiency depends on how it is integrated into the selection pipeline. When genomic prediction was applied after index construction, the responses were less consistent with the breeding objective, likely because selection acted on an already aggregated criterion. In contrast, performing genomic prediction before index construction preserved trait-specific genetic information before aggregation, allowing the index to better reflect the intended direction of selection for each trait. This was particularly evident for the Pesek-Baker index, which appeared to provide a more suitable framework for balancing gain in GY, reduction in CR, and maintenance of PH. Also, by clarifying these trade-offs, this study contributes to ongoing efforts to design breeding programs that are both efficient and sustainable. The results complement recent advances in multivariate GS models (Rutkoski et al. 2016), which may combine the advantages of trait-specific and index-based predictions. Additionally, genomic prediction performance in this study should be interpreted in the context of the fixed marker density used (540 SNPs). Because prediction accuracy depends on the linkage disequilibrium between markers and causal loci, alternative marker densities could affect the magnitude of the responses observed here. Future work should expand these simulations to include broader trait sets and to conduct empirical validation in real breeding populations, ensuring that GS–SI integration can be tailored effectively across different crops and environments.

## Supplementary Information

Below is the link to the electronic supplementary material.

Supplementary Material 1

Supplementary Material 2

## Abbreviations

GS: Genomic Selection)
SI: Selection Index
EMP: Empirical Index
SH: Smith–Hazel Index
PB: Pesek–Baker Index
TRAD: Traditional phenotypic selection
GY: Grain Yield
CR: Chalkiness Rate
PH: Plant Height
QTL: Quantitative Trait Locus/Loci
GV: Genetic Value
PM: Population Mean
Va: Additive Variance
h²: Heritability
SSD: Single Seed Descent
RR-BLUP: Ridge Regression Best Linear Unbiased Prediction
FDR: False Discovery Rate
MaCS: Markovian Coalescent Simulator
AlphaSimR: R package for breeding program simulations
