# Supplementary material for: Combining genomic prediction and multi-trait indices through stochastic simulations: do index type and deployment order affect genetic gain?
Source: Theor Appl Genet. 2026 Sep 27;139(10):284. doi: 10.1007/s00122-026-05398-0 (PMC13616857; doi:10.1007/s00122-026-05398-0)
Supplement: Supplementary file 1 [file 122_2026_5398_MOESM1_ESM.pdf]

```
############################################
# Simulating GS-based Multi-trait Breeding
# Roberto Fritsche-Neto and Jesimiel da Silva Viana
# roberto.neto@ncsu.edu
# Latest update: June 01, 2026
############################################
rm(list = ls())
Sys.time()
require(AlphaSimR)
require(MASS)
require(foreach)
require(doParallel)
# turn the warnings off
options(warn=-1)
# setting the number of cores that will be used
registerDoParallel(cores = detectCores()-6) # type the number of cores you want
to use
getDoParWorkers()

# Parameters
nQTL <- 360
segSites <- 1644
chip.size.1 <- 540
replicates <- 100
cycles <- 10
r <- 1:replicates
nChr <- 12

# Heritabilities by breeding stage (F2, F3, ..., F10)
h2_matrix <- matrix (
  c(0.05, 0.15, 0.45, 0.50, 0.55, 0.60, 0.65, 0.70, 0.75, #GY
    0.20, 0.25, 0.30, 0.35, 0.40, 0.45, 0.50, 0.55, 0.60, #CR
    0.16, 0.25, 0.40, 0.55, 0.60, 0.70, 0.85, 0.80, 0.90),#PH
  nrow = 3, byrow = TRUE
)

# Correlation among traits
corMatrix <- matrix(c(
  1.0,  0.10,  0.32,
  0.10, 1.0,  -0.20,
  0.32, -0.20, 1.0
), nrow = 3, byrow = TRUE)
colnames(corMatrix) <- rownames(corMatrix) <- c("GY", "CR", "PH")

# Crossing block
n.parents <- 40
n.crosses <- 80
progenie.size <- 100

################################################################################
########################## Crop history of evolution
###########################
################################################################################
```

```
set.seed(1234)

history <- runMacs(nInd = 1000,
                   nChr = nChr,
                   segSites = segSites / 12,
                   inbred = TRUE,
                   species = "GENERIC",
                   split = NULL,
                   ploidy = 2L)

SP <- SimParam$new(history)
#SP$nThreads = 1L

# add all traits together - more realistic regarding pleitripy and LD, but not
number the QTL
# considering the trade-off, this is the best option
SP$addTraitA(nQtlPerChr = nQTL / nChr,
              mean = c(0, 0, 0),
              var = c(1, 1, 1),
              corA = corMatrix)

# Add chip SNP
SP$addSnpChip(chip.size.1 / nChr)

# Setting the heritability of the trait
SP$setVarE(h2 = h2_matrix[, 4])
print(SP$varE)

# First Population
firstPop <- newPop(rawPop = history, isDH = TRUE, simParam = SP)

################################################################################
######################## Founders with multi-traits
############################
################################################################################
set.seed(1234)

# Parameters
n.GY <- round(0.7 * n.parents)  # 70% based on grain yield
n.CR <- round(0.15 * n.parents) # 15% based on chalkiness
n.PH <- round(0.15 * n.parents) # 15% based on plant height

# Selection based on each trait
GY_sel <- selectInd(firstPop, nInd = n.GY, trait = 1, use = "pheno", gender =
"B",
                    selectTop = TRUE, simParam = SP)
CR_sel <- selectInd(firstPop, nInd = n.CR, trait = 2, use = "pheno", gender =
"B",
                    selectTop = TRUE, simParam = SP)
PH_sel <- selectInd(firstPop, nInd = n.PH, trait = 3, use = "pheno", gender =
"B",
                    selectTop = TRUE, simParam = SP)

# Combine the three selected populations using mergePops
```

```
popList <- list(GY_sel, CR_sel, PH_sel)  # Create a list of selected
populations
founders_combined <- mergePops(popList) # Combine populations into a single
population

# Remove duplicates using unique() for individuals' IDs
unique_ids <- !duplicated(founders_combined@id)
founders <- founders_combined[unique_ids]

sink('init_variances.txt')
cat("Founders_combined: variance additive", diag(varA(founders_combined)),
"\n")
sink()

################################################################################
####################### Simulating Burn-in (F2 to F6)
##########################
################################################################################
set.seed(29121983)
cat("Burn-in\n")

# Define selection intensities
nSelF6 <- 40
nSelF7 <- 1
# Start the Burn-in process with the founder population
pop.trad <- founders
perform <- data.frame()

# Define standardize function inside the loop
standardize <- function(values) {
    if (sd(values) > 0) {
      return((values - mean(values)) / sd(values))
    } else {
      return(rep(0, length(values)))  # Assign 0 if standard deviation is 0
    }
  }

# Define target plant height
target_PH <- 0  # (target height = 0 in standardized units)

# Burn-in cycles
  for (i in 1:3) {
    cat("Cycle:", i, "\n")

    f1 <- randCross(pop = pop.trad, nCrosses = n.crosses, nProgeny = 1,
simParam = SP)
    f2 <- self(pop = f1, nProgeny = progenie.size, simParam = SP)
    f3 <- self(pop = f2, nProgeny = 1, simParam = SP)
    f4 <- self(pop = f3, nProgeny = 1, simParam = SP)
    f5 <- self(pop = f4, nProgeny = 1, simParam = SP)
    f6 <- self(pop = f5, nProgeny = 1, simParam = SP)
    SP$setVarE(h2 = h2_matrix[, 5])

    # Standardize traits and calculate empirical index
```

```
    GY_std <- standardize(f6@pheno[, 1])
    CR_std <- standardize(f6@pheno[, 2])
    PH_std <- standardize(f6@pheno[, 3])
    PH_abs <- abs(PH_std - target_PH)
    PH_trans <- -1 * (PH_abs - 1)

    SI <- 0.70 * GY_std - 0.15 * CR_std + 0.15 * PH_trans
    top_indices <- order(SI, decreasing = TRUE)[1:nSelF6]
    f6sel <- f6[top_indices]

    # Update base population for the next cycle
    pop.trad <- f6sel

    # variety for evaluation
    variety <- f6[top_indices[1]]

    # Record performance (register variance for each trait)
    varA <- varA(pop.trad)
    perform <- rbind(perform,
                     data.frame(Cycle = i,
                                Trait = c("GY", "CR", "PH"),
                                varA = as.numeric(diag(varA)),
                                MeanG = as.numeric(meanG(pop.trad))))
  }

  # Visualize performance
  print(perform)

################################################################################
#################### Creating Train Set with 3 traits
##########################
################################################################################
cat("GS Training Set", "\n")

# Initial heritability for the Train Set (TS)
SP$setVarE(h2 = h2_matrix[, 4])  # Using F4 heritability for all traits

# Crosses to generate the Train Set
TS0 <- randCross(pop = pop.trad, nCrosses = 80, nProgeny = 100, simParam = SP)

# Creating a new population from the progenies
TS00 <- newPop(rawPop = TS0, simParam = SP)

# Creating fully inbred lines via DH
TS000 <- makeDH(pop = TS00, nDH = 1, useFemale = TRUE, simParam = SP)

# Adding marker effects for each trait separately
markers_GY <- RRBLUP2(pop = TS000, traits = 1, use = "pheno", snpChip = 1,
simParam = SP, maxIter = 1000)
markers_CR <- RRBLUP2(pop = TS000, traits = 2, use = "pheno", snpChip = 1,
simParam = SP, maxIter = 1000)
markers_PH <- RRBLUP2(pop = TS000, traits = 3, use = "pheno", snpChip = 1,
simParam = SP, maxIter = 1000)
```

```
# Creating EBVs separately for each trait in a Validation Set
Val_Set <- randCross(pop = pop.trad, nCrosses = 80, nProgeny = 100, simParam =
SP)  # 40 crosses with 50 progenies each
VS_GY <- setEBV(Val_Set, markers_GY, simParam = SP)
VS_CR <- setEBV(Val_Set, markers_CR, simParam = SP)
VS_PH <- setEBV(Val_Set, markers_PH, simParam = SP)

# Combining EBVs into a single object
Val_Set@ebv <- cbind(VS_GY@ebv, VS_CR@ebv, VS_PH@ebv)

# Checking the results
print(head(Val_Set@ebv))  # Should display EBVs for Trait1, Trait2, Trait3

# Calculate accuracies
accuracy_GY <- as.numeric(cor(Val_Set@gv[, 1], Val_Set@ebv[, 1]))  # GY
accuracy_CR <- as.numeric(cor(Val_Set@gv[, 2], Val_Set@ebv[, 2]))  # CR
accuracy_PH <- as.numeric(cor(Val_Set@gv[, 3], Val_Set@ebv[, 3]))  # PH

# Create a dataframe
results_C0 <- data.frame(
  Method = c("Trad_Empirical", "Trad_Smith-Hazel", "Trad_Pesek-Baker",
"GS_Smith-Hazel", "GS_Pesek-Baker", "Smith-Hazel_GS", "Pesek-Baker_GS"),
  rep = 1,
  cycle = 0,
  PM_GY = as.numeric(mean(pop.trad@gv[,1])),
  PM_CR = as.numeric(mean(pop.trad@gv[,2])),
  PM_PH = as.numeric(mean(pop.trad@gv[,3])),
  Accuracy_GY = accuracy_GY,
  Accuracy_CR = accuracy_CR,
  Accuracy_PH = accuracy_PH,
  Var_GY = as.numeric(diag(varA(pop.trad))[1]),
  Var_CR = as.numeric(diag(varA(pop.trad))[2]),
  Var_PH = as.numeric(diag(varA(pop.trad))[3]),
  Variety_GY = as.numeric(variety@gv[1]),
  Variety_CR = as.numeric(variety@gv[2]),
  Variety_PH = as.numeric(variety@gv[3])
)

# Saving the results
write.table(results_C0, "results_C0.txt")

###########################################################################
############## Simulating SSD Method with Empirical Index at F6
################
###########################################################################
cat("Scenario 1 - Running Traditional Method with SSD until F5 and Empirical
Index at F6\n")
Sys.time()
# Loop for replicates
output_final <- data.frame()
results <- foreach(k = r,
                   .packages = c("AlphaSimR"),
                   .combine = "rbind",
                   .multicombine = TRUE,
```

```
                    .errorhandling = "remove",
                    .verbose = T
) %dopar% {

  # Initialize base population for each replicate
    pop.base <- pop.trad  # Ensure 'pop.trad' is defined
    # Initialize an object to store all results
    out <- data.frame()

    # Loop for cycles
    for (i in 1:cycles) {

      # F1 -> F6
      f1 <- randCross(pop = pop.base, nCrosses = n.crosses, nProgeny = 1,
simParam = SP)
      f2 <- self(pop = f1, nProgeny = progenie.size, simParam = SP)
      f3 <- self(pop = f2, nProgeny = 1, simParam = SP)
      f4 <- self(pop = f3, nProgeny = 1, simParam = SP)
      f5 <- self(pop = f4, nProgeny = 1, simParam = SP)
      f6 <- self(pop = f5, nProgeny = 1, simParam = SP)

      # Fix heritability for this cycle
      SP$setVarE(h2 = h2_matrix[, 5])

      # Apply Empirical Index
      GY_std <- standardize(f6@pheno[, 1])
      CR_std <- standardize(f6@pheno[, 2])
      PH_std <- standardize(f6@pheno[, 3])
      PH_abs <- abs(PH_std - target_PH)
      PH_trans <- -1 * (PH_abs - 1)

      SI <- 0.70 * GY_std - 0.15 * CR_std + 0.15 * PH_trans
      top_indices <- order(SI, decreasing = TRUE)[1:nSelF6]
      f6sel <- f6[top_indices]

      # Update base population for the next cycle
      pop.base <- f6sel

      # variety for evaluation
      variety <- f6[top_indices[1]]

      # Create a dataframe
      out <- rbind(out, data.frame(
        Method = "Trad_Empirical",
        rep = k,
        cycle = i,
        PM_GY = as.numeric(mean(pop.base@gv[,1])),
        PM_CR = as.numeric(mean(pop.base@gv[,2])),
        PM_PH = as.numeric(mean(pop.base@gv[,3])),
        Accuracy_GY = as.numeric(sqrt(diag(varA(pop.base))
[1]/diag(varP(pop.base))[1])),
        Accuracy_CR = as.numeric(sqrt(diag(varA(pop.base))
[2]/diag(varP(pop.base))[2])),
        Accuracy_PH = as.numeric(sqrt(diag(varA(pop.base))
```

```
[3]/diag(varP(pop.base))[3])),
        Var_GY = as.numeric(diag(varA(pop.base))[1]),
        Var_CR = as.numeric(diag(varA(pop.base))[2]),
        Var_PH = as.numeric(diag(varA(pop.base))[3]),
        Variety_GY = as.numeric(variety@gv[1]),
        Variety_CR = as.numeric(variety@gv[2]),
        Variety_PH = as.numeric(variety@gv[3])
        ))
    }
    output_final <- rbind(output_final, out)
  }

# saving results
index_name <- "Trad_Empirical"
output_file <- paste0("results_", index_name , ".txt")
write.table(results, output_file)

################################################################################
############### Simulating SSD Method with Smith-Hazel Index
###################
################################################################################
cat("Scenario 2 - Running SSD Simulation with Smith-Hazel Index\n")
Sys.time()
# Loop for replicates
output_final <- data.frame()
results <- foreach(k = r,
                   .packages = c("AlphaSimR"),
                   .combine = "rbind",
                   .multicombine = TRUE,
                   .errorhandling = "remove",
                   .verbose = T
) %dopar% {

  # Initialize base population for each replicate
  pop.base <- pop.trad  # Ensure 'pop.trad' is defined
  # Initialize an object to store all results
  out <- data.frame()

    # Loop for cycles
    for (i in 1:cycles) {

      # Perform random crosses and generate F1
      f1 <- randCross(pop = pop.base, nCrosses = n.crosses, nProgeny = 1,
simParam = SP)
      f2 <- self(pop = f1, nProgeny = progenie.size, simParam = SP)
      f3 <- self(pop = f2, nProgeny = 1, simParam = SP)
      f4 <- self(pop = f3, nProgeny = 1, simParam = SP)
      f5 <- self(pop = f4, nProgeny = 1, simParam = SP)
      f6 <- self(pop = f5, nProgeny = 1, simParam = SP)

      # Apply Smith-Hazel Index at F6
      SP$setVarE(h2 = h2_matrix[, 5])  # Set heritability for F6
      econWt <- c(0.7, -0.15, 0.15)  # Weights for GY, CR, PH
      P <- cov(f6@pheno)  # Covariance phenotypic matrix
```

```
      G <- cov(f6@gv) # Covariance genotypic matrix
      b <- solve(P) %*% G %*% econWt  # Smith-Hazel weights
      selIndex <- as.vector(f6@pheno[, 1:3] %*% b) # Calculate selection index

      # Add index as a new trait in the population
      f6@pheno <- cbind(f6@pheno, selIndex)

      # Select based on the selection index
      f6sel <- selectInd(pop = f6, nInd = nSelF6, trait = ncol(f6@pheno),
simParam = SP)

      # Update base population for the next cycle
      pop.base <- f6sel

      # variety for evaluation
      variety <- selectInd(pop = f6sel, nInd = nSelF7, trait = ncol(f6@pheno),
simParam = SP)

      #Create a dataframe
      out <- rbind(out, data.frame(
      Method = "Trad_Smith-Hazel",
      rep = k,
      cycle = i,
      PM_GY = as.numeric(mean(pop.base@gv[,1])),
      PM_CR = as.numeric(mean(pop.base@gv[,2])),
      PM_PH = as.numeric(mean(pop.base@gv[,3])),
      Accuracy_GY = as.numeric(sqrt(diag(varA(pop.base))
[1]/diag(varP(pop.base))[1])),
      Accuracy_CR = as.numeric(sqrt(diag(varA(pop.base))
[2]/diag(varP(pop.base))[2])),
      Accuracy_PH = as.numeric(sqrt(diag(varA(pop.base))
[3]/diag(varP(pop.base))[3])),
      Var_GY = as.numeric(diag(varA(pop.base))[1]),
      Var_CR = as.numeric(diag(varA(pop.base))[2]),
      Var_PH = as.numeric(diag(varA(pop.base))[3]),
      Variety_GY = as.numeric(variety@gv[1]),
      Variety_CR = as.numeric(variety@gv[2]),
      Variety_PH = as.numeric(variety@gv[3])
))
    }
  output_final <- rbind(output_final, out)
}

# saving results
index_name <- "Trad_Smith-Hazel"
output_file <- paste0("results_", index_name , ".txt")
write.table(results, output_file)

################################################################################
############# Simulating SSD Method with Pesek-Baker Index at F6
###############
################################################################################
cat(" Scenario 3 - Running SSD Simulation with Pesek-Baker Index at F6\n")
Sys.time()
```

```
# Loop for replicates
output_final <- data.frame()
results <- foreach(k = r,
                   .packages = c("AlphaSimR"),
                   .combine = "rbind",
                   .multicombine = TRUE,
                   .errorhandling = "remove",
                   .verbose = T
) %dopar% {

  # Initialize base population for each replicate
  pop.base <- pop.trad  # Ensure 'pop.trad' is defined
  # Initialize an object to store all results
  out <- data.frame()

  # Loop for cycles
  for (i in 1:cycles) {

      # Perform random crosses and generate F1
      f1 <- randCross(pop = pop.base, nCrosses = n.crosses, nProgeny = 1,
simParam = SP)
      f2 <- self(pop = f1, nProgeny = progenie.size, simParam = SP)
      f3 <- self(pop = f2, nProgeny = 1, simParam = SP)
      f4 <- self(pop = f3, nProgeny = 1, simParam = SP)
      f5 <- self(pop = f4, nProgeny = 1, simParam = SP)
      f6 <- self(pop = f5, nProgeny = 1, simParam = SP)
      # F6 generation
      SP$setVarE(h2 = h2_matrix[, 5])  # Set heritability for F6

      # Apply Pesek-Baker Index
      Y <- f6@pheno  # Retrieve phenotypic values
      desired_gains <- c(GY = 0.7, CR = -0.15, PH = 0.15)  # Customize desired
gains
      G_matrix <- cor(pheno(f6))   # Define the "G_matrix" based on phenotypes
      PB_weights <- solve(G_matrix) %*% desired_gains  # Define weights
      selIndex_PB <- as.vector(scale(Y) %*% PB_weights) # Calculate selection
index

      # Add index as a new trait in the population
      f6@pheno <- cbind(f6@pheno, selIndex_PB)

      # Select based on the selection index
      f6sel <- selectInd(pop = f6, nInd = nSelF6, trait = ncol(f6@pheno),
simParam = SP)

      # Update base population for the next cycle
      pop.base <- f6sel

      # variety for evaluation
      variety <- f6[top_indices[1]]

      #Create a dataframe
      out <- rbind(out, data.frame(
        Method = "Trad_Pesek-Baker",
```

```
        rep = k,
        cycle = i,
        PM_GY = as.numeric(mean(pop.base@gv[,1])),
        PM_CR = as.numeric(mean(pop.base@gv[,2])),
        PM_PH = as.numeric(mean(pop.base@gv[,3])),
        Accuracy_GY = as.numeric(sqrt(diag(varA(pop.base))
[1]/diag(varP(pop.base))[1])),
        Accuracy_CR = as.numeric(sqrt(diag(varA(pop.base))
[2]/diag(varP(pop.base))[2])),
        Accuracy_PH = as.numeric(sqrt(diag(varA(pop.base))
[3]/diag(varP(pop.base))[3])),
        Var_GY = as.numeric(diag(varA(pop.base))[1]),
        Var_CR = as.numeric(diag(varA(pop.base))[2]),
        Var_PH = as.numeric(diag(varA(pop.base))[3]),
        Variety_GY = as.numeric(variety@gv[1]),
        Variety_CR = as.numeric(variety@gv[2]),
        Variety_PH = as.numeric(variety@gv[3])
      ))
  }
  output_final <- rbind(output_final, out)
}

# saving results
index_name <- "Trad_Pesek-Baker"
output_file <- paste0("results_", index_name , ".txt")
write.table(results, output_file)

###########################################################################
############### GS univariate, then, Smith-Hazel at F6 #################
###########################################################################
cat("Scenario 4 - Running GS Univariate with Smith-Hazel Index\n")
Sys.time()
# Loop for replicates

output_final <- data.frame()
results <- foreach(k = r,
                   .packages = c("AlphaSimR"),
                   .combine = "rbind",
                   .multicombine = TRUE,
                   .errorhandling = "remove",
                   .verbose = T
) %dopar% {

  # Initialize an object to store all results
  out <- data.frame()
    # Initialize base population
    pop.base <- pop.trad
    TS <- TS000
    popList <- list(TS)
    snps_GY <- markers_GY
    snps_CR <- markers_CR
    snps_PH <- markers_PH

    # Loop for cycles
```

```
    for (i in 1:cycles) {

      # F1 to F6 generations (SSD process)
      f1 <- randCross(pop.base, nCrosses = n.crosses, nProgeny = 1, simParam =
SP)
      f2 <- self(pop = f1, nProgeny = progenie.size, simParam = SP)
      f3 <- self(pop = f2, nProgeny = 1, simParam = SP)
      f4 <- self(pop = f3, nProgeny = 1, simParam = SP)
      f5 <- self(pop = f4, nProgeny = 1, simParam = SP)

      # Adjust heritability and variance
      SP$setVarE(h2 = h2_matrix[, 5])
      f6 <- self(pop = f5, nProgeny = 1, simParam = SP)

      # Apply EBVs to F6 using the calculated values
      f6_GY <- setEBV(f6, solution = snps_GY, simParam = SP)
      f6_CR <- setEBV(f6, solution = snps_CR, simParam = SP)
      f6_PH <- setEBV(f6, solution = snps_PH, simParam = SP)

      # Create a single EBV matrix
      f6_ebvs_combined <- cbind(ebv(f6_GY)[, 1],ebv(f6_CR)[, 1],ebv(f6_PH)[,
1])
      colnames(f6_ebvs_combined) <- c("GY_GEBV", "CR_GEBV", "PH_GEBV")

      # Apply the combined EBVs to the population
      f6@ebv <- f6_ebvs_combined

      #Apply Smith-Hazel  Index at F6
      econWt <- matrix(c(0.7, -0.15, 0.15), ncol = 1)# Economic weights for
selection index
      P <- cov(f6@ebv[, 1:3])  # Covariance phenotypic matrix
      G <- cov(f6@ebv[, 1:3]) #Covariance genotypic matrix
      lambda <- 1e-6
      b <- solve(P + diag(lambda, ncol(P))) %*% G %*% econWt
      selIndex_GEBV <- as.vector(f6@ebv[, 1:3] %*% b)#Calculate Smith-Hazel
selection index

      # Add selection index as a new trait
      f6@ebv <- cbind(f6@ebv, SH_index_GEBV = selIndex_GEBV)

      # Select the best individuals
      f6_final <- selectInd(f6, nInd = nSelF6, trait = ncol(f6@ebv), use =
"ebv", simParam = SP)

      # Update base population for the next cycle
      pop.base <- f6_final

      # variety for evaluation
      selIndex_pheno <- as.vector(f6_final@pheno[, 1:3] %*% b)
      f6_final@pheno <- cbind(f6_final@pheno[, 1:3], SH_index_pheno =
selIndex_pheno)
      variety <- selectInd(pop = f6_final, nInd = nSelF7, trait =
ncol(f6_final@pheno), use = "pheno", simParam = SP)
```

```
      # Updating the TS and marker effects
      TSi <- f5
      popList <- c(popList, TSi)

      if (length(popList) <= 3) {
        TS <- mergePops(popList)
      } else {
        TS <- mergePops(popList[(length(popList)-2):length(popList)])
      }

      # Re-estimate marker effects
      snps_GY <- RRBLUP2(pop = TS, traits = 1, use = "pheno", snpChip = 1,
simParam = SP, maxIter = 10)
      snps_CR <- RRBLUP2(pop = TS, traits = 2, use = "pheno", snpChip = 1,
simParam = SP, maxIter = 10)
      snps_PH <- RRBLUP2(pop = TS, traits = 3, use = "pheno", snpChip = 1,
simParam = SP, maxIter = 10)

      # Creating a consolidated dataframe with final results
      out <- rbind(out, data.frame(
        Method = "GS_Smith-Hazel",
        rep = k,
        cycle = i,
        PM_GY = as.numeric(mean(pop.base@gv[,1])),
        PM_CR = as.numeric(mean(pop.base@gv[,2])),
        PM_PH = as.numeric(mean(pop.base@gv[,3])),
        Accuracy_GY = cor(gv(pop.base)[,1], ebv(pop.base)[,1]),
        Accuracy_CR = cor(gv(pop.base)[,2], ebv(pop.base)[,2]),
        Accuracy_PH = cor(gv(pop.base)[,3], ebv(pop.base)[,3]),
        Var_GY = as.numeric(diag(varA(pop.base))[1]),
        Var_CR = as.numeric(diag(varA(pop.base))[2]),
        Var_PH = as.numeric(diag(varA(pop.base))[3]),
        Variety_GY = as.numeric(variety@gv[1]),
        Variety_CR = as.numeric(variety@gv[2]),
        Variety_PH = as.numeric(variety@gv[3])
      ))
    }
    output_final <- rbind(output_final, out)
}

# saving results
index_name <- "GS_Smith-Hazel"
output_file <- paste0("results_", index_name , ".txt")
write.table(results, output_file)

################################################################################
############## GS univariate, then, Pesek-Baker at F6 ##################
################################################################################
cat(" Scenario 5 - Running GS Univariate with Pesek-Baker Index\n")
Sys.time()
# Loop for replicates
output_final <- data.frame()
results <- foreach(k = r,
                   .packages = c("AlphaSimR"),
```

```
                    .combine = "rbind",
                    .multicombine = TRUE,
                    .errorhandling = "remove",
                    .verbose = T
) %dopar% {

  # Initialize an object to store all results
  out <- data.frame()
    # Initialize base population and train set
    pop.base <- pop.trad
    TS <- TS000
    popList <- list(TS)
    snps_GY <- markers_GY
    snps_CR <- markers_CR
    snps_PH <- markers_PH

    # Loop for cycles
    for (i in 1:cycles) {

      # F1 to F6 generations (SSD process)
      f1 <- randCross(pop.base, nCrosses = n.crosses, nProgeny = 1, simParam =
SP)
      f2 <- self(pop = f1, nProgeny = progenie.size, simParam = SP)
      f3 <- self(pop = f2, nProgeny = 1, simParam = SP)
      f4 <- self(pop = f3, nProgeny = 1, simParam = SP)
      f5 <- self(pop = f4, nProgeny = 1, simParam = SP)

      # Set heritability for F6
      SP$setVarE(h2 = h2_matrix[, 5])
      f6 <- self(pop = f5, nProgeny = 1, simParam = SP)

      # Apply EBVs to F6 using the calculated values
      f6_GY <- setEBV(f6, solution = snps_GY, simParam = SP)
      f6_CR <- setEBV(f6, solution = snps_CR, simParam = SP)
      f6_PH <- setEBV(f6, solution = snps_PH, simParam = SP)

      # Create a single EBV matrix
      f6_ebvs_combined <- cbind(GY_GEBV = ebv(f6_GY)[, 1],CR_GEBV = ebv(f6_CR)
[, 1],PH_GEBV = ebv(f6_PH)[, 1])

      # Apply the combined EBVs to the population
      f6@ebv <- f6_ebvs_combined

      # Apply Pesek-Baker Index at F6
      desired_gains <- matrix(c(0.7, -0.15, 0.15), ncol = 1)
      X_gebv <- f6@ebv[, 1:3, drop = FALSE]
      G_matrix <- cov(scale(X_gebv))
      lambda <- 1e-6
      PB_weights <- solve(G_matrix + diag(lambda, ncol(G_matrix))) %*%
desired_gains
      selIndex_PB_GEBV <- as.vector(X_gebv %*% PB_weights)


      # Add the index as a new trait in the population
```

```
      f6@ebv <- cbind(f6@ebv[, 1:3, drop = FALSE],PB_index_GEBV =
selIndex_PB_GEBV)

      # Select the best individuals
      f6_final <- selectInd(f6, nInd = nSelF6, trait = ncol(f6@ebv), use =
"ebv", simParam = SP)

      # Update base population for the next cycle
      pop.base <- f6_final

      # variety for evaluation
      Y_pheno <- f6_final@pheno[, 1:3, drop = FALSE]
      G_matrix_pheno <- cov(scale(Y_pheno))
      PB_weights_pheno <- solve(G_matrix_pheno + diag(lambda,
ncol(G_matrix_pheno))) %*% desired_gains
      selIndex_PB_pheno <- as.vector(scale(Y_pheno) %*% PB_weights_pheno)
      f6_final@pheno <- cbind(f6_final@pheno[, 1:3, drop =
FALSE],PB_index_pheno = selIndex_PB_pheno)

      # Select final variety based on phenotypic PB index
      variety <- selectInd(pop = f6_final, nInd = nSelF7, trait =
ncol(f6_final@pheno), use = "pheno", simParam = SP)

      # Updating the TS and marker effects
      TSi <- f5
      popList <- c(popList, TSi)

      if (length(popList) <= 3) {
        TS <- mergePops(popList)
      } else {
        TS <- mergePops(popList[(length(popList)-2):length(popList)])
      }

      # Re-estimate marker effects
      snps_GY <- RRBLUP2(pop = TS, traits = 1, use = "pheno", snpChip = 1,
simParam = SP, maxIter = 10)
      snps_CR <- RRBLUP2(pop = TS, traits = 2, use = "pheno", snpChip = 1,
simParam = SP, maxIter = 10)
      snps_PH <- RRBLUP2(pop = TS, traits = 3, use = "pheno", snpChip = 1,
simParam = SP, maxIter = 10)

      # Creating a dataframe with final results
      out <- rbind(out, data.frame(
      Method = "GS_Pesek-Baker",
      rep = k,
      cycle = i,
      PM_GY = as.numeric(mean(pop.base@gv[,1])),
      PM_CR = as.numeric(mean(pop.base@gv[,2])),
      PM_PH = as.numeric(mean(pop.base@gv[,3])),
      Accuracy_GY = cor(gv(pop.base)[,1], ebv(pop.base)[,1]),
      Accuracy_CR = cor(gv(pop.base)[,2], ebv(pop.base)[,2]),
      Accuracy_PH = cor(gv(pop.base)[,3], ebv(pop.base)[,3]),
      Var_GY = as.numeric(diag(varA(pop.base))[1]),
      Var_CR = as.numeric(diag(varA(pop.base))[2]),
```

```
      Var_PH = as.numeric(diag(varA(pop.base))[3]),
      Variety_GY = as.numeric(variety@gv[1]),
      Variety_CR = as.numeric(variety@gv[2]),
      Variety_PH = as.numeric(variety@gv[3])
      ))
    }
    output_final <- rbind(output_final, out)
}

# saving results
index_name <- "GS_Pesek-Baker"
output_file <- paste0("results_", index_name , ".txt")
write.table(results, output_file)

###########################################################################
########### Simulating SSD Method with Smith-Hazel Index, then GS at F6
########
###########################################################################
cat("Scenario 6 - Running SSD Simulation with Smith-Hazel Index + GS at F6\n")
Sys.time()
# Loop for replicates
output_final <- data.frame()
results <- foreach(k = r,
                   .packages = c("AlphaSimR"),
                   .combine = "rbind",
                   .multicombine = TRUE,
                   .errorhandling = "remove",
                   .verbose = T
) %dopar% {

  # Initialize an object to store all results
  out <- data.frame()
  # Initialize base population and train set
    pop.base <- pop.trad  # Base population for the replication
    TS <- TS000
    popList <- list(TS)

    # Apply Smith-Hazel Index again
    econWt <- c(0.7, -0.15, 0.15)  # Weights for GY, CR, PH
    lambda <- 1e-6
    Y_TS <- TS@pheno[, 1:3, drop = FALSE]
    P <- cov(Y_TS)  # Covariance phenotypic matrix
    G <- cov(gv(TS)[, 1:3, drop = FALSE])    # Covariance genotypic matrix
    b_current <- solve(P + diag(lambda, ncol(P))) %*% G %*% econWt # Smith-
Hazel weights
    SI_TS <- as.vector(Y_TS %*% b_current)

    # Add the index as a fourth trait in pheno
    TS_SH <- TS
    TS_SH@pheno <- cbind(TS_SH@pheno[, 1:3, drop = FALSE],SH_index = SI_TS)

    # Genomic Selection  on the Index
    snps <- RRBLUP2(pop = TS_SH, traits = 4, use = "pheno", snpChip = 1,
simParam = SP, maxIter = 10)
```

```
    # Loop for cycles
    for (i in 1:cycles) {

      # F1 to F6 (SSD Process)
      f1 <- randCross(pop.base, n.crosses, nProgeny = 1, simParam = SP)
      f2 <- self(f1, progenie.size, simParam = SP)
      f3 <- self(f2, nProgeny = 1, simParam = SP)
      f4 <- self(f3, nProgeny = 1, simParam = SP)
      f5 <- self(f4, nProgeny = 1, simParam = SP)

      # Adjust heritability for F6
      SP$setVarE(h2 = h2_matrix[, 5])
      f6 <- self(f5, nProgeny = 1, simParam = SP)

      # Add EBVs in the population
      f6 <- setEBV(pop = f6, solution = snps, simParam = SP)

      # Select individuals based on EBV of the index
      f6sel <- selectInd(pop = f6, nInd = nSelF6, trait = 1, use = "ebv",
simParam = SP)

      # update the base pop
      pop.base <- f6sel

      # Select the best variety based on the Smith-Hazel Index
      Y_f6 <- f6sel@pheno[, 1:3, drop = FALSE]
      SI_f6_pheno <- as.vector(Y_f6 %*% b_current)
      f6sel@pheno <- cbind( f6sel@pheno[, 1:3, drop = FALSE],SH_index_pheno =
SI_f6_pheno)
      variety <- selectInd(pop = f6sel, nInd = nSelF7, trait =
ncol(f6sel@pheno), use = "pheno", simParam = SP)

      # updating the TS
      TSi <- f6
      popList <- c(popList, TSi)
      if (length(popList) <= 3) {
        TS <- mergePops(popList)
      } else {
        TS <- mergePops(popList[(length(popList) - 2):length(popList)])
      }

      # updating the marker effects - apply Smith-Hazel Index again
      Y_TS_int <- TS@pheno[, 1:3, drop = FALSE]
      P.int <- cov(Y_TS_int)
      G.int <- cov(gv(TS)[, 1:3, drop = FALSE])
      b.int <- solve(P.int + diag(lambda, ncol(P.int))) %*% G.int %*% econWt
      SI.int <- as.vector(Y_TS_int %*% b.int)
      TS_intern <- TS
      TS_intern@pheno <- cbind(TS_intern@pheno[, 1:3, drop = FALSE],SH_index =
SI.int)

      # Update marker effects for the Smith-Hazel index
      snps <- RRBLUP2(pop = TS_intern,traits = 4,use = "pheno",snpChip =
```

```
1,simParam = SP,maxIter = 10)
      b_current <- b.int

      # Store results in a DataFrame
      out <- rbind(out, data.frame(
        Method = "Smith-Hazel_GS",
        rep = k,
        cycle = i,
        PM_GY = as.numeric(mean(pop.base@gv[,1])),
        PM_CR = as.numeric(mean(pop.base@gv[,2])),
        PM_PH = as.numeric(mean(pop.base@gv[,3])),
        Accuracy_GY = as.numeric(cor(f6@ebv, gv(f6)[,1])),
        Accuracy_CR = as.numeric(cor(f6@ebv, gv(f6)[,2])),
        Accuracy_PH = as.numeric(cor(f6@ebv, gv(f6)[,3])),
        Var_GY = as.numeric(diag(varA(pop.base))[1]),
        Var_CR = as.numeric(diag(varA(pop.base))[2]),
        Var_PH = as.numeric(diag(varA(pop.base))[3]),
        Variety_GY = as.numeric(variety@gv[1]),
        Variety_CR = as.numeric(variety@gv[2]),
        Variety_PH = as.numeric(variety@gv[3])
      ))
    }
    output_final <- rbind(output_final, out)
}

# saving results
index_name <- "Smith-Hazel_GS"
output_file <- paste0("results_", index_name , ".txt")
write.table(results, output_file)

###########################################################################
######### Simulating SSD Method with Pesek-Baker Index, then GS at F6
##########
###########################################################################
cat("Scenario 7 - Running SSD Simulation with Pesek-Baker Index + GS at F6\n")
Sys.time()
#Loop for replicates
output_final <- data.frame()
results <- foreach(k = r,
                   .packages = c("AlphaSimR"),
                   .combine = "rbind",
                   .multicombine = TRUE,
                   .errorhandling = "remove",
                   .verbose = T
) %dopar% {

  # Initialize an object to store all results
  out <- data.frame()
  # Initialize base population and train set
    pop.base <- pop.trad  # Base population for replicate
    TS <- TS000
    popList <- list(TS)

    # Apply Pesek-Baker index at TS
```

```
    Y <- TS@pheno[, 1:3, drop = FALSE]
    desired_gains <- matrix(c(0.7, -0.15, 0.15), ncol = 1) # Define breeding
goals
    G_matrix <- cov(scale(Y)) #Define G_matrix covariance
    lambda <- 1e-6
    PB_weights <- solve(G_matrix + diag(lambda, ncol(G_matrix))) %*%
desired_gains

    # Calculate weights
    selIndex_PB <- as.vector(scale(Y) %*% PB_weights)

    # Add the index as a fourth trait in the pheno
    TS_PB <- TS
    TS_PB@pheno <- cbind(TS_PB@pheno[, 1:3, drop = FALSE], PB_index =
selIndex_PB)

    # Genomic Selection only to the Pesek-Baker Index
    snps <- RRBLUP2(pop = TS_PB,traits = 4,use = "pheno",snpChip = 1,simParam =
SP,maxIter = 10)

    for (i in 1:cycles) {

      # # F1 to F6 generations (SSD process)
      f1 <- randCross(pop.base, n.crosses, nProgeny = 1, simParam = SP)
      f2 <- self(f1, progenie.size, simParam = SP)
      f3 <- self(f2, nProgeny = 1, simParam = SP)
      f4 <- self(f3, nProgeny = 1, simParam = SP)
      f5 <- self(f4, nProgeny = 1, simParam = SP)

      # Adjust heritability for F6
      SP$setVarE(h2 = h2_matrix[, 5])
      f6 <- self(f5, nProgeny = 1, simParam = SP)

      # Add EBVs in the population
      f6 <- setEBV(pop = f6, solution = snps, simParam = SP)

      # Select individuals based on EBV of the index
      f6sel <- selectInd(pop = f6, nInd = nSelF6, trait = 1, use = "ebv",
simParam = SP)

      # update the base pop
      pop.base <- f6sel

      # Select the best variety based on the PB Index
      Y_f6 <- f6sel@pheno[, 1:3, drop = FALSE]
      G_f6 <- cov(scale(Y_f6))
      PB_weights_f6 <- solve(G_f6 + diag(1e-6, ncol(G_f6))) %*% desired_gains
      SI_f6_pheno <- as.vector(scale(Y_f6) %*% PB_weights_f6)
      f6sel@pheno <- cbind(f6sel@pheno[, 1:3, drop = FALSE], PB_index_pheno =
SI_f6_pheno)
      variety <- selectInd( pop = f6sel,nInd = nSelF7, trait =
ncol(f6sel@pheno), use = "pheno", simParam = SP)

      # Updating the TS and marker effects
```

```
      TSi <- f6
      popList <- c(popList, TSi)

      if (length(popList) <= 3) {
        TS <- mergePops(popList)
      } else {
        TS <- mergePops(popList[(length(popList) - 2):length(popList)])
      }

      # updating the marker effects - apply PB Index again
      Y.int <- TS@pheno[, 1:3, drop = FALSE]
      G.int <- cov(scale(Y.int)) #Define G_matrix covariance
      PB_weights.int <- solve(G.int + diag(lambda, ncol(G.int))) %*%
desired_gains   # Calculate weights
      SI.int <- as.vector(scale(Y.int) %*% PB_weights.int)

      # Add the index as a fourth trait in pheno
      TS.int <- TS
      TS.int@pheno <- cbind(TS.int@pheno[, 1:3, drop = FALSE], PB_index =
SI.int)
      snps <- RRBLUP2(pop = TS.int, traits = 4, use = "pheno", snpChip = 1,
simParam = SP, maxIter = 10)


      # Store results in a Data Frame
      out <- rbind(out, data.frame(
        Method = "Pesek-Baker_GS",
        rep = k,
        cycle = i,
        PM_GY = as.numeric(mean(pop.base@gv[,1])),
        PM_CR = as.numeric(mean(pop.base@gv[,2])),
        PM_PH = as.numeric(mean(pop.base@gv[,3])),
        Accuracy_GY = as.numeric(cor(f6@ebv, gv(f6)[,1])),
        Accuracy_CR = as.numeric(cor(f6@ebv, gv(f6)[,2])),
        Accuracy_PH = as.numeric(cor(f6@ebv, gv(f6)[,3])),
        Var_GY = as.numeric(diag(varA(pop.base))[1]),
        Var_CR = as.numeric(diag(varA(pop.base))[2]),
        Var_PH = as.numeric(diag(varA(pop.base))[3]),
        Variety_GY = as.numeric(variety@gv[1]),
        Variety_CR = as.numeric(variety@gv[2]),
        Variety_PH = as.numeric(variety@gv[3])
      ))
    }
    output_final <- rbind(output_final, out)
}

# saving results
index_name <- "Pesek-Baker_GS"
output_file <- paste0("results_", index_name , ".txt")
write.table(results, output_file)
Sys.time()
############### the end ##################
```
