# Supplementary material for: Combining genomic prediction and multi-trait indices through stochastic simulations: do index type and deployment order affect genetic gain?
Source: Theor Appl Genet. 2026 Sep 27;139(10):284. doi: 10.1007/s00122-026-05398-0 (PMC13616857; doi:10.1007/s00122-026-05398-0)
Supplement: Supplementary file 2 [file 122_2026_5398_MOESM2_ESM.pdf]

```r
#########################################
# Graph - updating methods
########################################
rm(list = ls()) # clear the environment

# packages
library("tidyr")
library('dplyr')
library("ggplot2")
library('ggpubr')
library('gridExtra')
library(data.table)

# loading data
outputs <- dir()[dir() %like% "results"]
list.results <- list()
for(i in 1:length(outputs)){
  list.results[[i]] <- read.table(outputs[i])
}

data <- do.call(rbind, list.results)
str(data)
head(data)
dim(data)
tail(data)
unique(data$Method)
data$Method <- as.factor(data$Method)
data$length <- ifelse(data$Method %like% "GS", 3, 5)
data$year <- data$cycle*data$length


# Graphs
theme_sim <- theme(
  axis.title = element_text(size = 6, face = "bold"),
  axis.title.x = element_blank(),
  axis.text = element_text(size = 5),
  strip.text = element_text(size = 6, face = "bold"),
  legend.position = "bottom",
  legend.text = element_text(size = 5),
  legend.title = element_text(size = 6, face = "bold"),
  legend.key.height = unit(3, "mm")
)

make_plot <- function(data, trait, ylab){

  data %>%
    group_by(Method, year) %>%
    summarise(
      value = mean(.data[[trait]], na.rm = TRUE),
      .groups = "drop"
    ) %>%
```

```r
    ggplot(aes(year, value, colour = Method)) +
    geom_line(linewidth = 0.5) +
    geom_point(size = 1) +
    scale_x_continuous(
      limits = c(0, 30),
      breaks = seq(0, 30, 5)
    ) +
    labs(y = ylab) +
    theme_sim
}


traits <- c(
  "PM_GY", "PM_CR", "PM_PH",
  "Accuracy_GY", "Accuracy_CR", "Accuracy_PH",
  "Var_GY", "Var_CR", "Var_PH"
)

ylabs <- c(
  "Population mean for GY",
  "Population mean for CR",
  "Population mean for PH",
  "Selection accuracy for GY",
  "Selection accuracy for CR",
  "Selection accuracy for PH",
  "Va for GY",
  "Va for CR",
  "Va for PH"
)

plots <- Map(
  \(trait, lab) make_plot(data, trait, lab),
  traits,
  ylabs
)


g <- ggarrange(
  plotlist = plots,
  ncol = 3,
  nrow = 3,
  common.legend = TRUE,
  legend = "right",
  labels = letters[1:9],
  font.label = list(size = 8)
)

g

# saving graph
ggsave(filename = './Fig2.png',
```

```
       plot = g,
       device = 'png',
       width = 174,
       height = 130,
       units = 'mm',
       dpi = 300)



#### ggstatsplot
set.seed(123)
library(ggstatsplot)
head(data)

df_data <- dplyr::filter(data, year == 30)
dim(df_data)
head(df_data)

library(WRS2) # for data
# Sort Method factor levels by mean PM_GY
df_data2 <- df_data %>%
  group_by(Method) %>%
  mutate(Method = factor(Method, levels = names(sort(tapply(PM_GY, Method,
mean, rm.na = T), decreasing = TRUE))))


p1 <- ggwithinstats(as.data.frame(df_data2), Method, PM_GY,
                    pairwise.comparisons = T,
                    type = "robust",
                    results.subtitle = F,
                    ylab = "PM for GY after 30 years") +
  theme(axis.text.x = element_text(angle = 45, hjust = 1))

p2 <- ggwithinstats(as.data.frame(df_data2), Method, PM_CR,
                    pairwise.comparisons = T,
                    type = "robust",
                    results.subtitle = F,
                    ylab = "PM for CR after 30 years") +
  theme(axis.text.x = element_text(angle = 45, hjust = 1))

p3 <- ggwithinstats(as.data.frame(df_data2), Method, PM_PH,
                    pairwise.comparisons = T,
                    type = "robust",
                    results.subtitle = F,
                    ylab = "PM for PH after 30 years") +
  theme(axis.text.x = element_text(angle = 45, hjust = 1))


p.final <- ggstatsplot::combine_plots(
  plotlist = list(p1, p2, p3))
```

```
ggsave(filename = './Fig3.png',
       plot = p.final,
       device = 'png',
       width = 300,
       height = 130,
       units = 'mm',
       dpi = 300)


##### pairwise non-parametric comparisons ###########
head(df_data2)
library(data.table)
df_data2$GS_or_TRAD <- df_data2$Method %like% "GS"
df_data2$GS_or_TRAD <- ifelse(df_data2$GS_or_TRAD == T, "GS", "TRAD")

# Create boxplot with Kruskal-Wallis test result shown
p4 <- ggstatsplot::ggbetweenstats(
  data = df_data2,
  x = GS_or_TRAD,
  y = PM_GY,
  ylab = "PM for GY after 30 years",
  xlab = "GS or TRAD based breeding",
  type = "nonparametric",  # Automatically chooses Kruskal-Wallis for >2
groups
  pairwise.comparisons = TRUE,
  pairwise.display = "significant",  # show only significant comparisons
  p.adjust.method = "BH",            # adjust p-values
  messages = FALSE
)

p5 <- ggstatsplot::ggbetweenstats(
  data = df_data2,
  x = GS_or_TRAD,
  y = PM_CR,
  ylab = "PM for CR after 30 years",
  xlab = "GS or TRAD based breeding",
  type = "nonparametric",  # Automatically chooses Kruskal-Wallis for >2
groups
  pairwise.comparisons = TRUE,
  pairwise.display = "significant",  # show only significant comparisons
  p.adjust.method = "BH",            # adjust p-values
  messages = FALSE
)

# Create boxplot with Kruskal-Wallis test result shown
p6 <- ggstatsplot::ggbetweenstats(
  data = df_data2,
  x = GS_or_TRAD,
  y = PM_PH,
  ylab = "PM for PH after 30 years",
  xlab = "GS or TRAD based breeding",
```

```
  type = "nonparametric",  # Automatically chooses Kruskal-Wallis for >2
groups
  pairwise.comparisons = TRUE,
  pairwise.display = "significant",  # show only significant comparisons
  p.adjust.method = "BH",            # adjust p-values
  messages = FALSE
)

p.final2 <- ggstatsplot::combine_plots(
  plotlist = list(p4, p5, p6))

ggsave(filename = './Fig4.png',
       plot = p.final2,
       device = 'png',
       width = 300,
       height = 130,
       units = 'mm',
       dpi = 300)


#### among SI
df_data2$SI <- as.character(df_data2$Method)
df_data2$SI[df_data2$SI %like% "Smith"] <- "Smith-Hazel"
df_data2$SI[df_data2$SI %like% "Pesek"] <- "Pesek-Baker"
df_data2$SI[df_data2$SI %like% "Empirical"] <- "Empirical"

# Create boxplot with Kruskal-Wallis test result shown
p7 <- ggstatsplot::ggbetweenstats(
  data = df_data2,
  x = SI,
  y = PM_GY,
  ylab = "PM for GY after 30 years",
  xlab = "Selection index",
  type = "nonparametric",  # Automatically chooses Kruskal-Wallis for >2
groups
  pairwise.comparisons = TRUE,
  pairwise.display = "significant",  # show only significant comparisons
  p.adjust.method = "BH",            # adjust p-values
  messages = FALSE
)

p8 <- ggstatsplot::ggbetweenstats(
  data = df_data2,
  x = SI,
  y = PM_CR,
  ylab = "PM for CR after 30 years",
  xlab = "Selection index",
  type = "nonparametric",  # Automatically chooses Kruskal-Wallis for >2
groups
  pairwise.comparisons = TRUE,
  pairwise.display = "significant",  # show only significant comparisons
```

```
  p.adjust.method = "BH",            # adjust p-values
  messages = FALSE
)


p9 <- ggstatsplot::ggbetweenstats(
  data = df_data2,
  x = SI,
  y = PM_PH,
  ylab = "PM for PH after 30 years",
  xlab = "Selection index",
  type = "nonparametric",  # Automatically chooses Kruskal-Wallis for >2
groups
  pairwise.comparisons = TRUE,
  pairwise.display = "significant",  # show only significant comparisons
  p.adjust.method = "BH",            # adjust p-values
  messages = FALSE
)


p.final3 <- ggstatsplot::combine_plots(
  plotlist = list(p7, p8, p9))

ggsave(filename = './Fig5.png',
       plot = p.final3,
       device = 'png',
       width = 300,
       height = 130,
       units = 'mm',
       dpi = 300)

###### GS before or after?
head(df_data2)
dim(df_data2)
df_data3 <- df_data2[df_data2$Method %like% "GS",]
dim(df_data3)
df_data3$BorA <- ifelse(df_data3$Method %like% "GS_", "Before", "After")

p10 <- ggstatsplot::ggbetweenstats(
  data = df_data3,
  x = BorA,
  y = PM_GY,
  ylab = "PM for GY after 30 years",
  xlab = "GS before or after the SI",
  type = "nonparametric",  # Automatically chooses Kruskal-Wallis for >2
groups
  pairwise.comparisons = TRUE,
  pairwise.display = "significant",  # show only significant comparisons
  p.adjust.method = "BH",            # adjust p-values
  messages = FALSE
)
```

```
p11 <- ggstatsplot::ggbetweenstats(
  data = df_data3,
  x = BorA,
  y = PM_CR,
  ylab = "PM for CR after 30 years",
  xlab = "GS before or after the SI",
  type = "nonparametric",  # Automatically chooses Kruskal-Wallis for >2
groups
  pairwise.comparisons = TRUE,
  pairwise.display = "significant",  # show only significant comparisons
  p.adjust.method = "BH",            # adjust p-values
  messages = FALSE
)

p12 <- ggstatsplot::ggbetweenstats(
  data = df_data3,
  x = BorA,
  y = PM_PH,
  ylab = "PM for PH after 30 years",
  xlab = "GS before or after the SI",
  type = "nonparametric",  # Automatically chooses Kruskal-Wallis for >2
groups
  pairwise.comparisons = TRUE,
  pairwise.display = "significant",  # show only significant comparisons
  p.adjust.method = "BH",            # adjust p-values
  messages = FALSE
)

p.final4 <- ggstatsplot::combine_plots(
  plotlist = list(p10, p11, p12))

ggsave(filename = './Fig6.png',
       plot = p.final4,
       device = 'png',
       width = 300,
       height = 130,
       units = 'mm',
       dpi = 300)

### the end ###
```
